# High-intensity interval training versus moderate-intensity continuous training on patient quality of life in cardiovascular disease: a systematic review and meta-analysis

**DOI:** 10.1038/s41598-023-40589-5

**Published:** 2023-08-25

**Authors:** Haohan Yu, Xudong Zhao, Xiaoxia Wu, Jing Yang, Jun Wang, Lijuan Hou

**Affiliations:** 1 College of P.E and Sports, Beijing Normal University, Beijing, China; 2 Faculty of Psychology, Beijing Normal University, Beijing, China; 3 State Key Laboratory of Cognitive Neuroscience and Learning, Beijing Normal University, Beijing, China; 4 Department of respiratory and critical care medicine, First Affiliated Hospital of Fujian Medical University, Fuzhou, China

**Keywords:** Quality of life, Rehabilitation

## Abstract

This systematic review and meta-analysis aimed to compare the effects of high-intensity interval training (HIIT) and moderate-intensity continuous training (MICT) on the quality of life (QOL) and mental health (MH) of patients with cardiovascular disease (CVDs). Web of Science, Medline, Embase, Cochrane (CENTRAL), CINAHL, China National Knowledge Infrastructure, Wanfang, and China Science and Technology Journal databases were searched from their date of establishment to July, 2023. A total of 5798 articles were screened, of which 25 were included according to the eligibility criteria. The weighted mean difference (WMD) and standardized mean difference (SMD) were used to analyze data from the same and different indicator categories, respectively. The fixed-effects model (FE) or random-effects model (RE) combined data based on the between-study heterogeneity. There were no statistically significant differences regarding QOL, physical component summary (PCS), mental component summary (MCS), and MH, including depression and anxiety levels, between the HIIT and MICT groups [SMD = 0.21, 95% confidence interval (CI)  − 0.18–0.61, Z = 1.06, P = 0.290; SMD = 0.10, 95% CI  − 0.03–0.23, Z = 1.52, P = 0.128; SMD = 0.07, 95% CI  − 0.05–0.20, Z = 1.13, P = 0.25; SMD = − 0.08, 95% CI  − 0.40–0.25, Z = − 0.46, P = 0.646; WMD = 0.14. 95% CI  − 0.56–0.84, Z = 0.39, P = 0.694, respectively]. HIIT significantly improved PCS in the coronary artery disease (CAD) population subgroup relative to MICT. HIIT was also significantly superior to MICT for physical role, vitality, and social function. We conclude that HIIT and MICT have similar effects on QOL and MH in patients with CVD, while HIIT is favorable for improving patients’ self-perceived physiological functioning based on their status and social adjustment, and this effect is more significant in patients with CAD.

## Introduction

Cardiovascular diseases (CVDs) are the leading cause of death worldwide, and the prevalence and mortality rates of CVDs continue to rise worldwide. From 1990 to 2019, the number of people living with cardiovascular disease in 204 countries worldwide has increased from 271 to 523 million; a growth rate of 92.99%, and the number of deaths has risen from 12.1 million to 18.6 million, according to data published by the Global Burden of Disease Study 2019 (GBD2019). To date, CVDs have been among the top 10 diseases for all-cause mortality worldwide^1^. CVD broadly refers to a group of diseases caused by abnormalities of the circulatory system, including coronary artery disease (CAD), hypertension, angina pectoris (AP), heart failure (HF), myocardial infarction (MI), and stroke. These diseases can have a serious impact on an individual’s health status, and so, effective interventions and rehabilitations are essential^2^.

Although a large number of studies have reported various beneficial management strategies, such as surgery, medication, and nursing care, for the physiological function of patients with CVDs, limited focus has been placed on patients’ quality of life (QOL) and mental health (MH). The World Health Organization (WHO) categorizes health into three main elements: physical health, mental health, and social adjustment, and defines a synergistic relationship in which all three elements complement each other in health promotion^3^. Of these, MH and social adaptation can be included in QOL, which is defined as “the experience of individuals living in different cultures and value systems with respect to their goals, expectations, standards, and conditions of existence that are linked to their concerns”^4^. In clinical studies, QOL can generally be assessed using scales, such as the MOS 36-item short form health survey (SF-36) or the MacNew, which provide a multidimensional and comprehensive assessment of the patient’s rehabilitation status through the physical component summary (PCS), mental component summary (MCS), and eight sub-indicators related to social adjustment^5^. For patients with CVDs, QOL is an important indicator for assessing the degree of recovery and effectiveness of treatment during care and return visits due to the multiplicity and depth of indicators involved^6^. Unlike objective measures, such as blood pressure and heart rate, QOL outcomes are based on patients’ subjective perceptions. Therefore, by observing QOL, physicians and caregivers can have a clearer understanding of the development of the patient’s condition and changes in the patient’s proprioception, which is of great significance for the adjustment of nursing care measures and development of rehabilitation.

Cardiac rehabilitation (CR) is an important measure of secondary care for CVDs and aims to help patients improve the efficiency of rehabilitation, reduce the occurrence of cardiac risk events, lower the level of negative emotions, such as depression and anxiety, and improve QOL, to promote both physiological and psychological health^7^. CR involves measures such as health education, lifestyle behavior modification, psychosocial support, and supervised exercise programs. Physical activity (PA), an important tool, has been widely shown to induce positive cardiovascular adaptations in patients to improve QOL and MH^8^. Relative to other interventions, PA is superior for improving physiological and psychological health because regular movement of skeletal muscles mediates metabolic elevations and the release of chemicals in the brain related to mood^9,10^. Currently, PA is an important non-pharmacological therapy for the treatment of CVDs and has been recognized in major guidelines worldwide^8,11^.

Although a large body of evidence demonstrates the effectiveness of PA in CR, some studies have found that different types of PA may lead to differences in QOL versus MH during the patients’ return visits^12,13^. In early studies, the American College of Sports Medicine and the American Heart Association (AHA) generally recommended moderated-intensity continuous training (MICT) for patients with CVDs^11,14^. However, with the development of sports science, the AHA first introduced high-intensity interval training (HIIT) to the field of athletic training for CR in 2007^15^. HIIT consists of high intensity exercise interspersed with active recovery periods of medium-to-low intensity, with peak volume alternating with recovery intervals. Studies have shown that high-intensity exercise mediates a greater degree of physiological stimulation^16^. However, patients with CVDs remain highly sensitive to exercise, and due to their high cardiac risk factors, excessively high exercise intensity can place undue stress and burden on the cardiovascular system, leading to elevated blood pressure and increased risk of cardiac arrhythmias. Therefore, by improving training capacity, the AHA offers an interval training strategy performed with acceptable loads that ensures comparable benefits to cardiorespiratory fitness (CRF) to those associated with high-intensity exercise, while reducing the probability of cardiac risk events^15^. HIIT has been recognized as an effective exercise modality and has received widespread attention from the clinical field and healthcare professionals, with some European and North American athletic teams increasingly adopting HIIT protocols in CR^17,18^. It has been found that HIIT not only improves mitochondrial biogenesis, insulin sensitivity, glucose regulation, high-density lipoprotein cholesterol, deep abdominal adiposity, and blood pressure more than MICT, but also has more advantages in improving skeletal muscle strength, cardiorespiratory fitness, and athletic ability^19–21^.

A large number of meta-analyses have demonstrated that HIIT improves physiological parameters in patients with CVDs significantly more so than does MICT; however, it is unclear whether HIIT has a significant impact on cardiovascular outcomes^13,22,23^. Furthermore, the number of studies that have explored the differences between HIIT and MICT using QOL and HM as primary indicators is limited. During CR, psychological health is as important as physiological health, and both affect the patient’s QOL. In light of recent developments in the medical model, QOL is being widely used as a new indicator for health assessment. Therefore, this study is the first to evaluate QOL and MH, including depression and anxiety, as primary outcome indicators to explore the efficacy of HIIT and MICT in CR from the perspective of patient’s proprioception. This study also aimed to provide an effective program and theoretical reference for the rehabilitation and nursing care of CVDs based on PA. The hypotheses of this study were (1) HIIT is superior to MICT in improving QOL, and (2) HIIT is superior to MICT in reducing anxiety and depression.

## Results

### Search results

**Figure 1:**
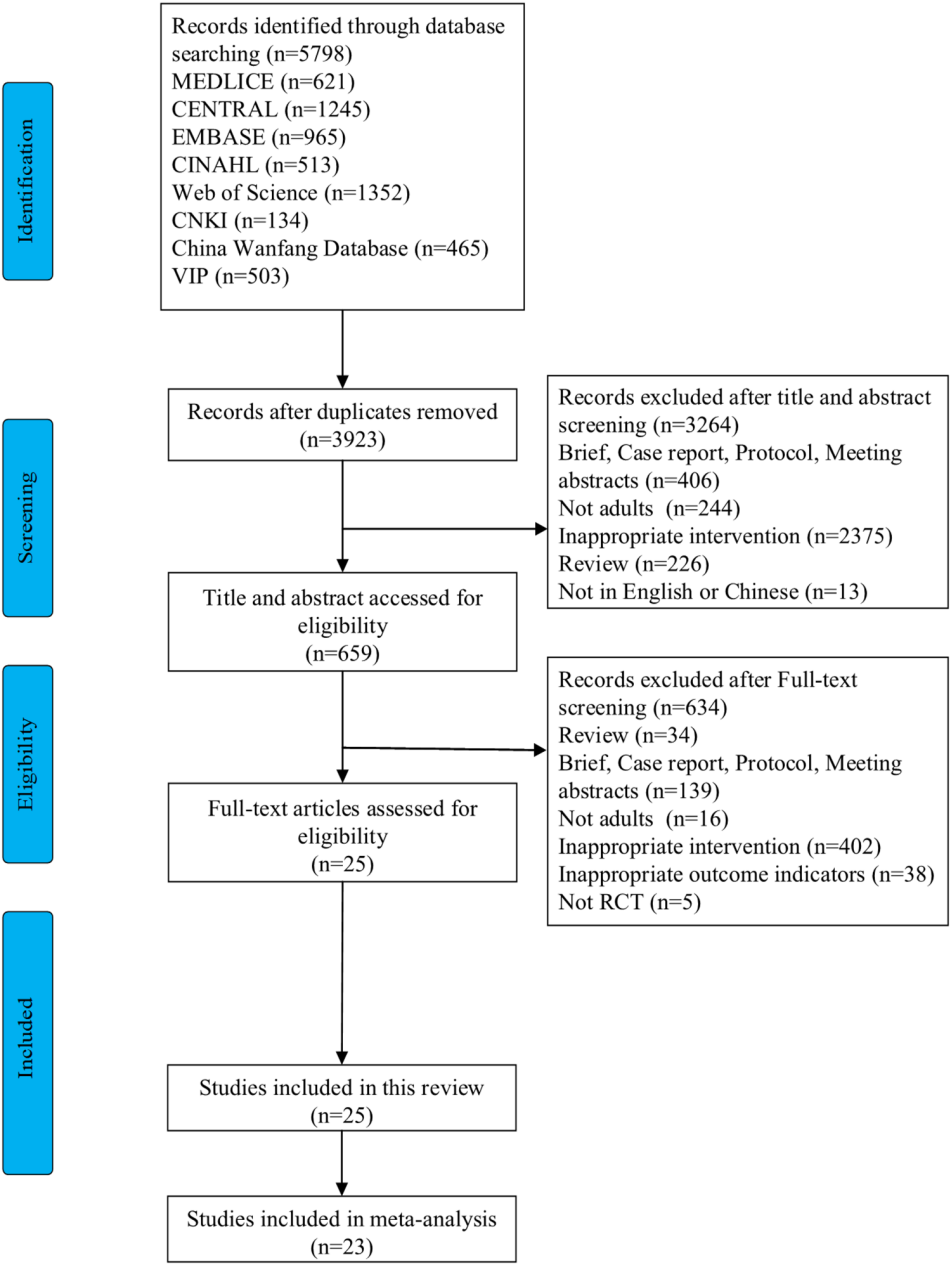
Flow diagram of the evaluation process.

Figure 1 displays a flowchart of the literature screening. A total of 5798 articles were retrieved, and after excluding duplicate literature using a literature management software, 3923 articles remained. A total of 659 articles were included in the preliminary screening of their title and abstract. After full-text screening, 633 articles were excluded, leaving 25 remaining. A total of 25 studies were included in this systematic review^12,24–46^, of which 23 were included in the meta-analysis^12,24–38,40–43,45,46^.

### Study characteristics

The characteristics of the included studies are summarized in Table 1. The included studies were from 12 different countries, with Canada^25,29,39,47^ and Norway^27,38,44,45^ dominating the origin of articles. All articles were written in English, except for one published in China that was written in Chinese^35^. Three studies included were randomized controlled trials^12,26,32^. The study participants were predominantly (11/25 studies) patients with coronary heart disease. The remaining studies involved patients with HF^24,26,38,43^, tetralogy of Fallot^12^, heart transplantation^27,36^, coronary artery bypass grafting^44^, atrial fibrillation^47^, myocardial infarction^34^, stroke^37^, and hypertension^35,41^. Most of the studies reported the registration numbers, while eight did not^25,26,32,34,42–44,46^.

**Table 1:** Characteristics of studies.

ID	Study	Country	Language	Study design	Participant	Simple size, n	Sex	Age	Trail registration
1	Anderson (2019)	Brazil	English	Single-blinded RCT	Patients with heart failure with preserved ejection fraction	HIIT = 10	M = 3/F = 7	60 ± 9	NCT02916225
						MICT = 9	M = 4/F = 5	60 ± 10	
2	Currie (2015)	Canada	English	RCT	Patients with coronary artery disease	HIIT = 9	M = 9/F = 0	63 ± 8	UN
						MICT = 10	M = 9/F = 1	66 ± 8	
3	Koufaki (2014)	England	English	Pilot RCT	Patients with heart failure with preserved ejection fraction	HIIT = 8	UN	UN	UN
						MICT = 9	UN	UN	
4	Novakovic (2018)	Slovenia	English	Pilot RCT	Patients with tetralogy of fallot	HIIT = 9	M = 2/F = 7	36.2 ± 6.8	NCT02643810
						MICT = 9	M = 4/F = 5	40.1 ± 10.4	
5	Nytroen (2019)	Norway	English	Multi-center, prospective, RCT	Heart transplant recipients	HIIT = 37	M = 28/F = 9	50 ± 12	NCT01796379
						MICT = 41	M = 29/F = 12	48 ± 14	
6	Okur (2020)	Turkey	English	RCT	Patients with coronary artery disease	HIIT(LV) = 6	M = 6/F = 0	61.83 ± 4.79	NCT04048057
						HIIT(HV) = 7	M = 7/F = 0	59.14 ± 3.63	
						MICT = 7	M = 5/F = 2	62.00 ± 6.61	
7	Reed (2021)	Canada	English	Single-centre, parallel-group, RCT	Patients with coronary artery disease	HIIT = 43	M = 36/F = 7	61 ± 7	NCT02765568
						MICT = 44	M = 38/F = 6	60 ± 7	
8	Schonfelder (2021)	Australia	English	RCT	Patients with coronary artery disease	HIIT = 17	UN	UN	NCT1493193
						MICT = 14	UN	UN	
9	Taylor (2020)	Australia	English	Single-center RCT	Patients with coronary artery disease	HIIT = 46	M = 39/F = 7	65 ± 7	ACTRN12615001292561
						MICT = 47	M = 39/F = 8	65 ± 8	
10	Villafaina (2020)	Spain	English	Pilot RCT	Patients with coronary artery disease	HIIT = 11	M = 11/F = 0	55.27 ± 7.13	UN
						MICT = 10	M = 10/F = 0	56.55 ± 3.50	
11	Yakut (2022)	Turkey	English	RCT	Patients with myocardial infarction	HIIT = 11	M = 10/F = 1	59.6 ± 4.5	NCT04407624
						MICT = 10	M = 8/F = 2	58.5 ± 5.6	
12	Xianghui Liu (2021)	China	Chinese	RCT	Patients with hypertension	HIIT = 13	M = 4/F = 9	44 ± 9	UN
						MICT = 13	M = 5/F = 8	48 ± 7	
13	Dall (2015)	Denmark	English	RCT	Heart transplant recipients	HIIT = 8	M = 12/F = 4	51.9 (33–70)	NTC01914406
						MICT = 9			
14	Chih-Chin Hsu (2020)	China	English	RCT	Stroke patients	HIIT = 10	M = 8/F = 2	58.5 (49.8–67.2)	NCT04135391
						MICT = 12	M = 12/F = 1	53.1 (46.2–60.0)	
15	Ellingsen (2017)	Norway	English	RCT	Patients with heart failure with preserved ejection fraction	HIIT = 90	M = 76/F = 14	65 (58–68)	NCT00917046
						MICT = 85	M = 73/F = 12	60 (58–65)	
16	Dunford (2021)	Canada	English	RCT	Patients with coronary artery disease	HIIT = 9	M = 8/F = 1	61 ± 8	NCT03235674
						MICT = 9	M = 8/F = 1	62 ± 6	
17	McGregor (2023)	England	English	Multi-center, prospective, RCT	Patients with coronary artery disease	HIIT = 187	M = 176/F = 11	58.6 ± 9.2	NCT02784873
						MICT = 195	M = 180/F = 15	59 ± 9.9	
18	Koldobika (2016)	Spain	English	Prospective, RCT	Patients with ischemic heart disease	HIIT = 36	M = 28/F = 8	58 ± 11	NCT02168712
						MICT = 36	M = 33/F = 3	58 ± 11	
19	Mikel (2020)	Spain	English	RCT	Patients with hypertension	HIIT(LV) = 65	M = 43/F = 22	54.7 ± 7.2	NCT02283047
						HIIT(HV) = 60	M = 39/F = 21	53.0 ± 8.6	
						MICT = 61	M = 38/F = 23	54.2 ± 7.2	
20	Pattyn (2016)	Belgium	English	Multi-center, prospective, RCT	Patients with coronary artery disease	HIIT = 80	M = 76/F = 4	57.4 ± 8.7	UN
						MICT = 83	M = 76/F = 7	59.9 ± 9.2	
21	Papathanasiou (2020)	Bulgaria	English	Prospective, RCT	Patients with chronic heart failure	HIIT = 60	M = 35/F = 7	63.65 ± 6.71	UN
						MICT = 60	M = 76/F = 7	63.82 ± 6.71	
22	Reed (2022)	Canada	English	RCT	Patients with atrial fibrillation	HIIT = 43	M = 29/F = 14	68 ± 8	NCT02602457
						MICT = 43	M = 28/F = 15	71 ± 7	
23	Trine (2009)	Norway	English	RCT	Patients undergoing coronary artery bypass grafting	HIIT = 33	M = 24/F = 4	60.2 ± 6.9	UN
						MICT = 36	M = 24/F = 7	62.0 ± 7.6	
24	Ulrik (2007)	Norway	English	RCT	Patients with stable postinfarction heart failure	HIIT = 9	M = 7/F = 2	76.5 ± 9	NCT00218933
						MICT = 9	M = 7/F = 2	74.4 ± 12	
25	Viviane (2014)	Belgium	English	Prospective, RCT	Patients with coronary artery disease	HIIT = 100	M = 91/F = 9	57.0 ± 8.8	UN
						MICT = 100	M = 89/F = 11	59.9 ± 9.2	

### Exercise intervention characteristics

Table 2 shows the characteristics of the HIIT and MICT exercise programs. The narrative summaries of the exercise interventions are as follows: With the exception of 5 studies that did not mention the intervention sites^12,25,27,35,40^, 3 were conducted at home^31,34,44^, 14 at the hospital and rehabilitation center^24,26,28,29,33,36–39,42–44,46,47^, and the remaining studies were conducted the firm, university, or indoor tennis court^30,32,41^. Exercise was conducted under supervision in all studies except two^34,41^, and three studies did not report information regarding supervision^12,28,33^. Two studies did not report data on adherence to exercise^34,41^, while in the remaining studies, exercise adherence ranged 67.1–99.0%^12,24–33,35–40,42–46^. Adverse events during the exercise interventions were reported in 19 studies^12,24,27,29,31–34,36,38–40,42–47^ and of the two reported adverse exercise events^29,31^. In all but two studies^26,31^, warm-up or relaxation exercises were involved in the exercise program. The frequency of exercise was mainly 2–3 days per week, the exercise duration ranged 20–65 min, and the intervention duration ranged 4–52 weeks.

**Table 2:** Exercise intervention characteristics.

ID	Study	Setting	Supervised	Adherence	Adverse events	Additional stages	Interventions	Model	Intensity	Duration	Frequency	Time	Outcomes
1	Anderson (2019)	Hospital	YES	97%	NO	10 min warm-up, 3 min cool-down	HIIT: high intensity sprint for 4 min, active recovery for 3 min, alternating 4 sets	Treadmill	80–90% VO_2max_, 85–95% HRmax, 15–17 in RPE	38 min	3 days/week	12 weeks	MLHF
				95%		/	MICT: moderate intensity running all the time		50–60% VO_2max_, 60–70% HRmax, 11–13 in RPE	47 min			
2	Currie (2015)	UN	YES	92%	UN	10 min warm-up and cool-down	HIIT: high intensity cycling for 1 min, moderate intensity cycling for 1 min, alternating 10 sets	Cycle ergometer	85% PPO alternating 10% PPO for mouth 1, 100% PPO for month 2, 108% PPO for month 3, 121% PPO for final 3 months	20 min	2 days/week	24 weeks	SF-36
				90%			MICT: moderate intensity cycling all the time		57% PPO for month 1–3, 78% PPO for final 3 months	30 min at start, add 10 min monthly to 50 min			
3	Koufaki (2014)	Hospital	YES	90%	UN	/	HIIT: high intensity cycling for 30 s, moderate intensity cycling for 1 min, alternating 20 sets	Cycle ergometer	100% PPO alternating 20–30% PPO	30 min	3 days/week	24 weeks	MLHF, SF-36
							MICT: moderate intensity cycling all the time		40–60% VO_2max_	20-30 min increasing to 40 min			
4	Novakovic (2018)	UN	UN	92.90%	NO	5 min warm-up and cool-down	HIIT: high intensity cycling for 1 min, moderate intensity cycling for 3 min, alternating 8 sets	Cycle ergometer	80% HR_max_ alternating 60% HRmax at start, add 5% HRmax after 12th and 24th	42 min	2–3 days/week	12–18 weeks	SF-36
				89.20%		8 min warm-up, 7 min cool-down	MICT: moderate intensity cycling all the time		70% HRmax at start, add 5% HRmax after 12th and 24th	41 min			
5	Nytroen (2019)	UN	YES	81%	NO	10 min warm-up, 5 min cool-down	HIIT: high intensity exercise for 4 min, active recovery for 3 min, alternating 4 sets	Resistance training	85–95% HRmax, 81–93% VO_2max_, 16–18 in RPE	40 min	2–3 days/week	36 weeks	SF-36, HADS
							MICT: moderate intensity exercise all the time	Core strengthening exercise	60–80% MEC	40 min			
6	Okur (2020)	Hospital	UN	99%	UN	10 min warm-up and cool-down	HIIT(LV): high intensity exercise for 1 min, active recovery for 1 min, alternating 10 sets	UN	85–100% W_max_ alternating 50–70% W_max_	30 min	5 days/week	24 sessions	SF-36, MacNew
							HIIT(HV): high intensity exercise for 4 min, active recovery for 3 min, alternating 4 sets			38 min			
							MICT: moderate intensity exercise all the time		50–70% W_max_	20 min increasing to 30 min			
7	Reed (2021)	University of Ottawa heart institute	YES	73.80%	YES	10 min warm-up, 5-10 min cool-down	HIIT: high intensity exercise for 4 min alternating low intensity exercise for 3 min	Treadmill, cycle ergometer, elliptical	85–95% HRmax alternating 60–70% HRmax	45 min	2 days/week	12 weeks	SF-36, BDI
				76.30%		10-15 min warm-up, 15 min cool-down	MICT: aerobic exercise all the time		20-40 bpm above HRrest, 12–16 in REP	60 min			
8	Schonfelder (2021)	Ergoline GmbH, Bitz	YES	99.20%	UN	5 min warm-up and cool-down	HIIT: high intensity exercise for 4 min, active recovery for 3 min, alternating 4 sets	Resistance training	85–95% HRmax alternating 60–70% HRmax	25 min	3 days/week	6 weeks	MacNew, HADS
							MICT: moderate intensity exercise all the time	Endurance training	65–85% HRmax	33 min			
9	Taylor (2020)	Hospital, home	YES	92.50%	YES	/	HIIT: high intensity exercise for 4 min, active recovery for 3 min, alternating 4 sets	Treadmill, cycle ergometer, elliptical	85–95% HRmax, 15–18 alternating 11–13 in RPE	32 min	3 days/week	52 weeks	MacNew
							MICT: moderate intensity exercise all the time		65–75% HRmax, 11–13 in RPE	60 min			
10	Villafaina (2020)	Indoor tennis court	YES	100%	NO	8 min warm-up, 10 min cool-down	HIIT: high intensity exercise for 15 s, 4 sets of low intensity exercise for 15 s following 3 sets of active recovery for 1 min	Tennis	85–90% HRmax alternating 50% HRmax	60 min	3 days/week	12 weeks	SF-36
				90%			MICT: moderate intensity exercise all the time		70–85% HRmax	60 min			
11	Yakut (2022)	Home	NO	UN	NO	10 min warm-up and cool-down	HIIT: high intensity running for 4 min alternating low intensity running for 3 min	Walking	85–95% HRmax or 15–18 in RPE alternating 70–75% HRmax or < 14 in RPE	45 min	2 days/week	12 weeks	MacNew
							MICT: moderate intensity running all the time		70–75% HRmax, 12–14 in RPE	40-65 min			
12	Xianghui Liu (2021)	UN	YES	95.80%	UN	5 min warm-up and cool-down	HIIT: high intensity cycling for 1 min alternating low intensity cycling for 2 min	Cycle ergometer	80% HRmax alternating 50% HRmax	50 min	3 days/week	16 weeks	SF-36
							MICT: moderate intensity cycling all the time		60% HRmax	50 min			
13	Dall (2015)	The heart centre, rigshospitalet, copenhagen	YES	94.11%	NO	10 min warm-up and cool-down	HIIT: high intensity cycling for 4 or 2 min, active recovery for 2 min, alternating 8 sets	Cycle ergometer	80% HRmax alternating 60% HRmax	30 min	3 days/week	12 weeks	SF-36, HADS
							MICT: moderate intensity cycling all the time		60–70% HRmax	45 min			
14	Chih-Chin Hsu (2020)	Hospital	YES	76.92%	UN	3 min warm-up and cool-down	HIIT: high intensity cycling for 3 min, moderate intensity cycling for 3 min, alternating 5 sets	Cycle ergometer	80% VO_2max_ alternating 40% VO_2max_	30 min	2–3 days/week	36 sessions	SF-36
				86.67%			MICT: moderate intensity cycling all the time		60% VO_2max_	30 min			
15	Ellingsen (2017)	Hospital	YES	85.56%	YES	Moderate intensity warm-up and cool-down	HIIT: high intensity exercise for 4 min, active recovery for 3 min, alternating 4 sets	Treadmill, cycle ergometer	90–95% HRmax alternating active recovery	38 min	3 days/week	12 weeks	KCCQ, HADS, GMS, DS14
				76.47%		/	MICT: moderate intensity exercise all the time		60–70% HRmax	47 min			
16	Dunford (2021)	Hospital	YES	100 ± 105%	NO	10 min warm-up, 5 min cool-down	HIIT: continuously ascending and descending the stairs six times (12 steps), active recovery for 90 s, alternating 3 sets	Stair climbing	20 alternating 14–15 in RPE	3 sets	6 sessions in hospital, 3 sessions/week in home	12 weeks	QLMI
				104 ± 75%			MICT: moderate intensity exercise all the time	Treadmill, cycle ergometer, walking	60–80% HRR, 11–13 in RPE	30 min			
17	McGregor (2023)	UN	YES	72.72%	YES	10-15 min warm-up	HIIT: high intensity cycling for 1 min, moderate intensity cycling for 1 min, alternating 10 sets	Cycle ergometer, walking	85–90% PPO alternating 20–25% PPO	10 sets	2 days/week	8 weeks	EQ-5D
				78.97%			MICT: moderate intensity exercise all the time		40–70% HRR, 60–80% MEC	20-40 min			
18	Koldobika (2016)	Hospital	YES	92%	NO	12, 10, 7 min warm-up, 13, 10, 8 min cool-down for week 1–3, 5 min warm-up and cool-down for final 5 weeks	HIIT: high intensity cycling for 20 s, moderate intensity cycling for 40 s, alternating 15 sets at first, add 5 sets weekly	Cycle ergometer	50% MEC alternating 10%MEC	40 min	3 days/week	8 weeks	SF-36, MacNew
				87.50%			MICT: moderate intensity cycling all the time		VT1 for week 1–4, VT1 + 10% for week 5–8	40 min			
19	Mikel (2020)	University of Basque Country	YES	UN	UN	5-10 min warm-up, 10 min cool-down	HIIT(LV): high intensity exercise for 30 s, moderate intensity exercise for 90 s, alternating 9 sets	Cycling, walking, swimming	90% VO_2max_ alternating 65% VO_2max_	20 min	5–7 days/week	16 weeks	SF-36
							HIIT(HV): high intensity exercise for 30 s, moderate intensity exercise for 90 s, alternating 18 sets		90% VO_2max_ alternating 65% VO_2max_	45 min			
							MICT: aerobic continuous exercise all the time		65% VO_2max_	45 min			
20	Pattyn (2016)	Hospital	YES	85%	YES	10 min warm-up	HIIT: high intensity cycling for 4 min, active recovery for 3 min, alternating 4 sets	Cycle ergometer	85–95% HRmax alternating 50–70% HRmax	38 min	3 days/week	12 weeks	SF-12
				89%		5 min warm-up and cool-down	MICT: moderate intensity cycling all the time		70–75% HRmax	47 min			
21	Papathanasiou (2020)	Center of rehabilitation and sports medicine	YES	93.75%	NO	10 min warm-up, 5 min cool-down	HIIT: high intensity exercise alternating moderate intensity exercise for 3 sets	Body-weight training	90% HRmax alternating 70% HRmax	40 min	2 days/week	12 weeks	MLHF
				88.23%		/	MICT: moderate intensity cycling all the time	Cycle ergometer	70% HRmax	40 min			
22	Reed (2022)	Center of rehabilitation and sports medicine	YES	76.19%	YES	2 min warm-up, 1 min cool-down	HIIT: high intensity cycling for 30 s, active recovery for 30 s, alternating 20 sets	Cycle ergometer	80–100% PPO alternating active recovery	23 min	2 days/week	12 weeks	SF-36
				92.85%		10-15 min warm-up, 15 min cool-down	MICT: aerobic continuous exercise all the time	Cycling, walking, elliptical, rowing	UN	60 min			
23	Trine (2009)	Center of rehabilitation and sports medicine, home	YES	84.84%	NO	8 min warm-up, 5 min cool-down	HIIT: high intensity running for 4 min, active recovery for 3 min, alternating 4 sets	Treadmill	90% HRmax alternating active recovery	41 min	5 days/week	4 weeks	MacNew
				86.11%		/	MICT: moderate intensity running all the time		70% HRmax	46 min			
24	Ulrik (2007)	Hospital, home	YES	92 ± 2%	NO	10 min warm-up, 3 min cool-down	HIIT: high intensity running for 4 min alternating active recovery for 3 min	Treadmill, outdoor uphill walking	90–95% HRmax alternating 50–70% HRmax	38 min	3 days/week	12 weeks	MacNew
				95 ± 3%		/	MICT: moderate intensity running all the time		70–75% HRmax	47 min			
25	Viviane (2014)	Hospital	YES	85%	NO	10 min warm-up	HIIT: high intensity cycling for 4 min, active recovery for 3 min, alternating 4 sets	Cycle ergometer	85–90% VO_2max_, 90–95% HRmax, alternating 60–70% HRmax	38 min	3 days/week	12 weeks	SF-12
				89%		5 min warm-up, 5 min cool-down	MICT: moderate intensity cycling all the time		60–70% VO_2max_, 65–75% HRmax	47 min			

*UN* unclear, *NO* no mention, *RPE* rating of perceived exertion, *HR*max maximal heart rate, *VO2*max maximal oxygen consumption, *VT* ventilatory threshold, *PPO* peak power output, *MLHF* Minnesota living with heart failure questionnaire, *SF-36* the MOS 36-item short-form health survey, *SF-12* the MOS 12-item short-form health survey, *MacNew* the MacNew heart disease health related quality of life instrument, *KCCQ* the Kansas city cardiomyopathy questionnaire, *DS14* type D personality scale-14, *GMS* geriatric mental status scale, *QLMI* the quality of life after myocardial infarction questionnaire, *EQ-5D* the EuroQOL instrument, *HADS* hospital anxiety and depression scale, *BDI* back depression inventory.

### Methodological quality

**Figure 2:**
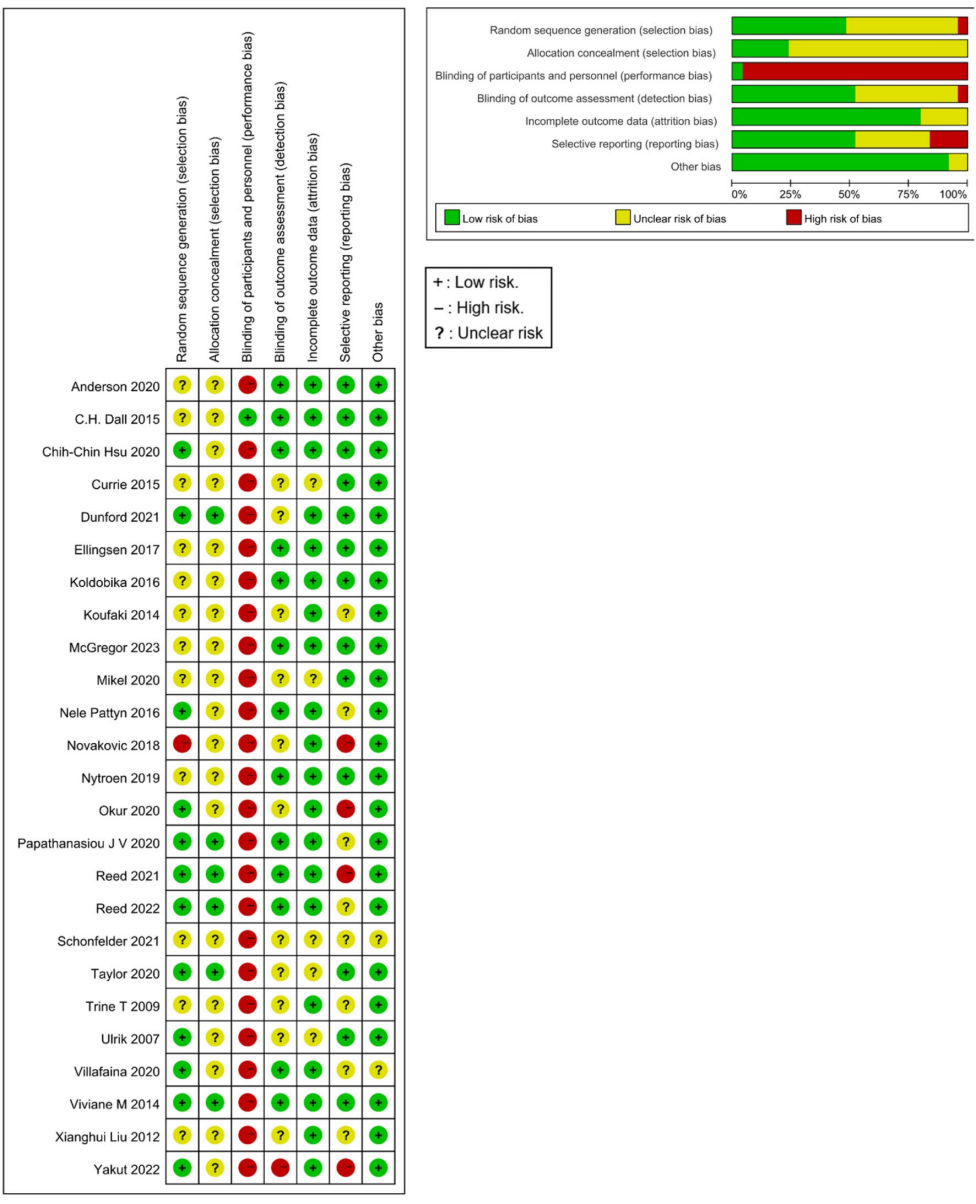
Risk of bias summary.

The details of the quality assessment are summarized in Fig. 2. In the quality assessment, three differences appeared between researchers regarding two studies^31,33^, involving random sequence generation, assignment hiding, and blind setting of outcome evaluators. All of these issues were resolved after discussion among researchers. The consistency among the evaluators was as follows: Kappa value, 0.961; weighted Kappa value, 0.968; and intraclass correlation coefficient, 0.979 (two-way random, absolute agreement). However, there were some methodological flaws among blinded participants and interventionists, and these studies were rated as having a high risk of bias.

### GRADE of evidence

According to the Grading of Recommendations and Assessment Development and Evaluation (GRADE) evidence summary, the certainty of QOL, physical component summary (PCS), mental component summary (MCS), and anxiety was moderate, but the certainty of depression was low. Detailed results are shown in Supplement 2.

### Outcomes

#### QOL

**Figure 3:**
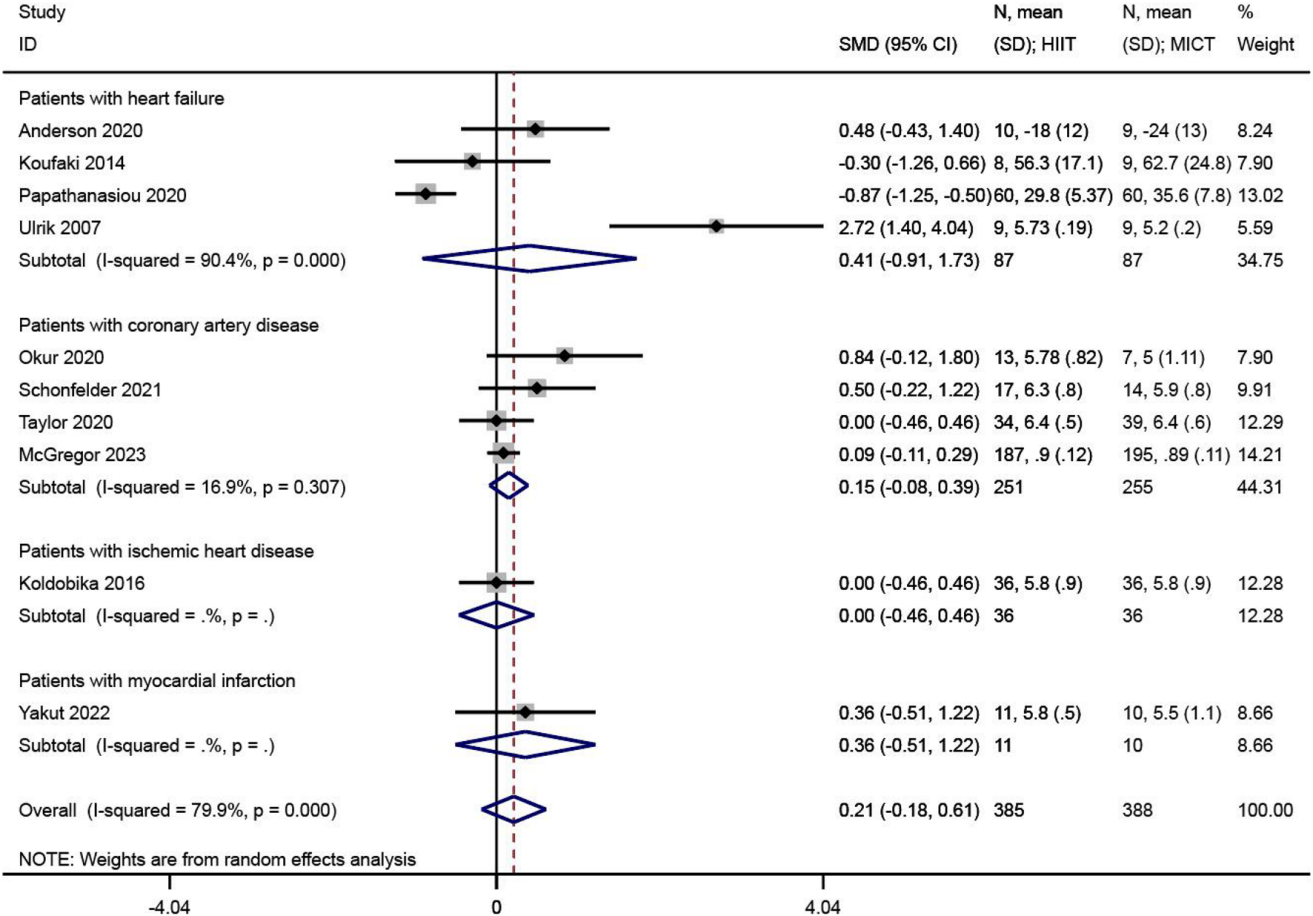
The results of meta-analyses of the effect of quality of life. The results of HIIT and MICT are shown as: mean, standard deviation (sample size).

QOL was reported in 10 of the 23 included studies^24,26,28,30,31,33,34,40,43,45^. The effect size synthesis was performed using the standardized mean difference (SMD) in the meta-analysis. There was significant heterogeneity between studies (I^2^ = 79.9%, P < 0.001). The RE analysis showed that the difference in QOL levels between the HIIT and MICT groups was not statistically significant (SMD = 0.21, 95% CI  − 0.18 ~ 0.61, Z = 1.06, P = 0.290), as shown in Fig. 3.

#### PCS

**Figure 4:**
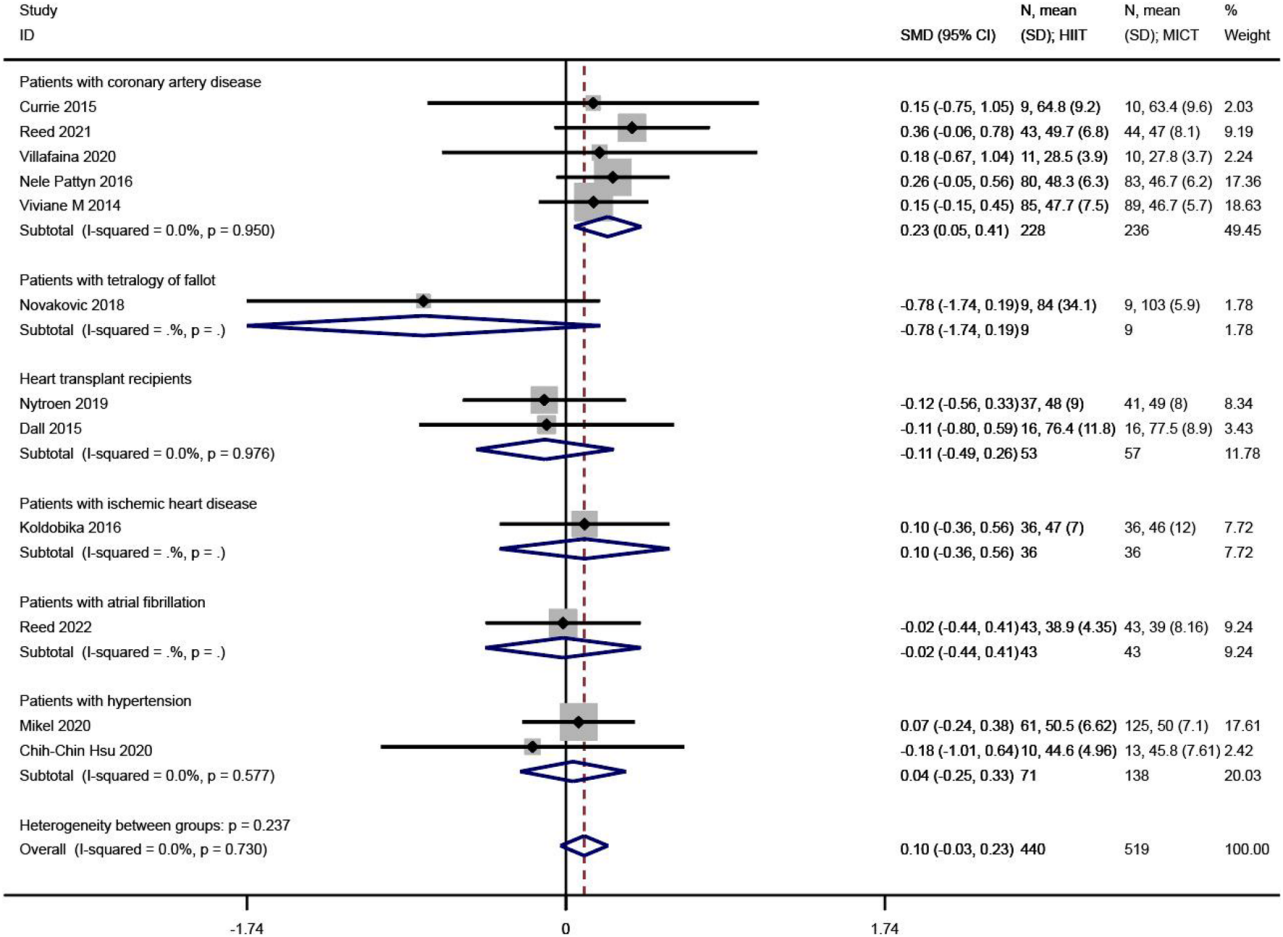
The results of meta-analyses of the effect of physical component summary. The results of HIIT and MICT are shown as: mean, standard deviation (sample size).

PCS levels were reported in 12 of the 23 studies^12,25,27,29,32,33,36,37,41,42,46,47^. The SMD was used in the meta-analysis for effect size synthesis, and no between-study heterogeneity was detected (I^2^ = 0.00%, P = 0.730). The FE analysis showed that the difference in PCS levels between the HIIT and MICT groups was not statistically significant (SMD = 0.10, 95% CI  − 0.03 ~ 0.23, Z = 1.52, P = 0.128). However, the subgroup analysis showed that, in patients with coronary heart disease, PCS was significantly higher in the HIIT group compared to the MICT group (SMD = 0.23, 95% CI  0.05 ~ 0.41, Z = 2.45, P = 0.014), as shown in Fig. 4.

#### MCS

**Figure 5:**
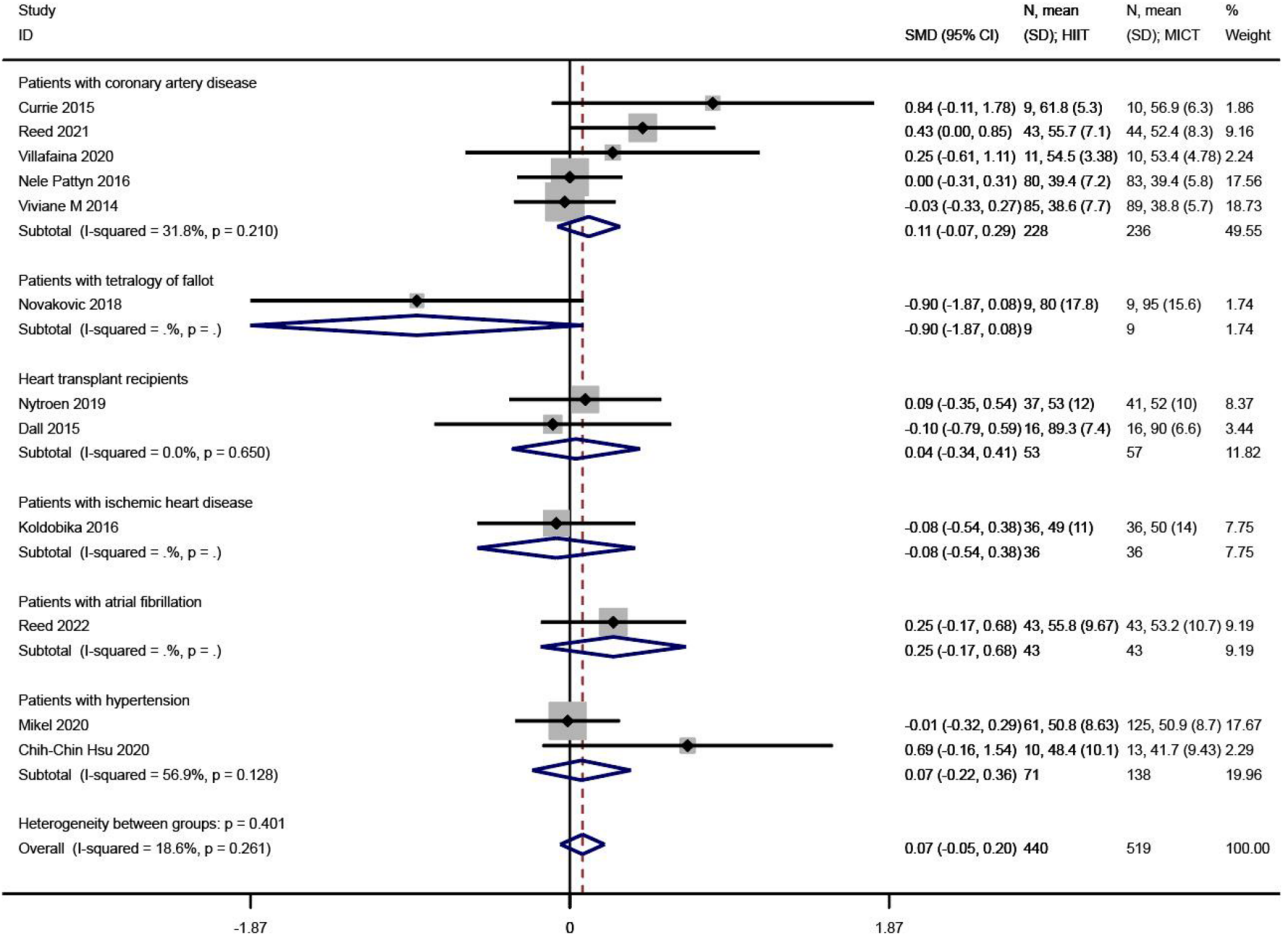
The results of meta-analyses of the effect of mental component summary. The results of HIIT and MICT are shown as: mean, standard deviation (sample size).

MCS levels were reported in 12 of the 23 studies^12,25,27,29,32,33,36,37,41,42,46,47^. The SMD was used in the meta-analysis for effect size synthesis, and low heterogeneity was observed between studies (I^2^ = 18.6%, P = 0.261). The FE analysis showed that the difference in MCS levels between the HIIT and MICT groups was not statistically significant (SMD = 0.07, 95% CI  − 0.05 ~ 0.20, Z = 1.13, P = 0.259), as shown in Fig. 5.

#### Depression

**Figure 6:**
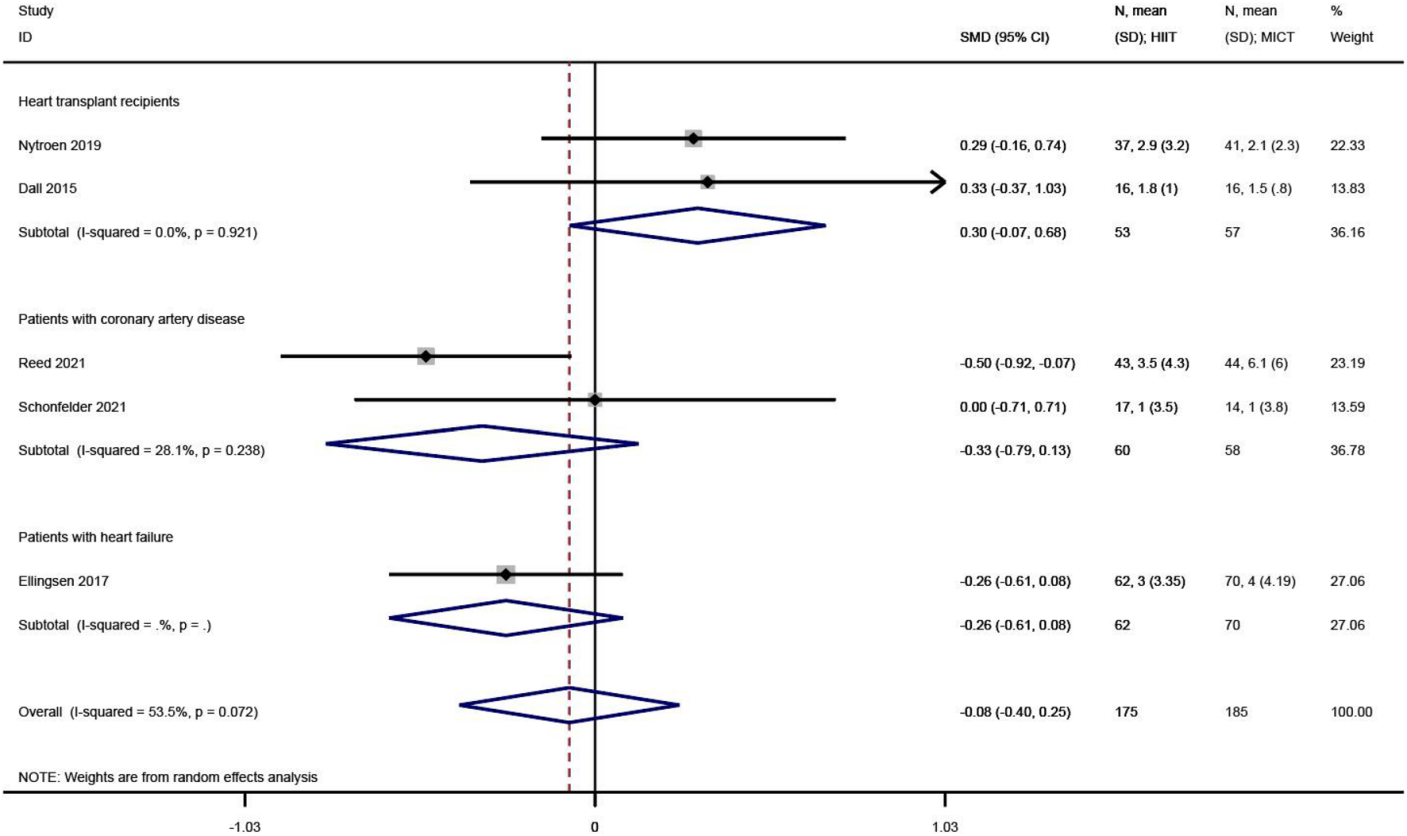
The results of meta-analyses of the effect of depression. The results of HIIT and MICT are shown as: mean, standard deviation (sample size).

Depression levels were reported in 5 of the 23 studies^27,29,30,36,38^. The SMD was used in the meta-analysis for effect size synthesis, and significant heterogeneity was detected between the studies (I^2^ = 53.5%, P = 0.072). The FE analysis showed that the difference in depression levels between the HIIT and MICT groups was not statistically significant (SMD = − 0.08, 95% CI  − 0.40 ~ 0.25, Z = − 0.46, P = 0.646), as shown in Fig. 6.

#### Anxiety

**Figure 7:**
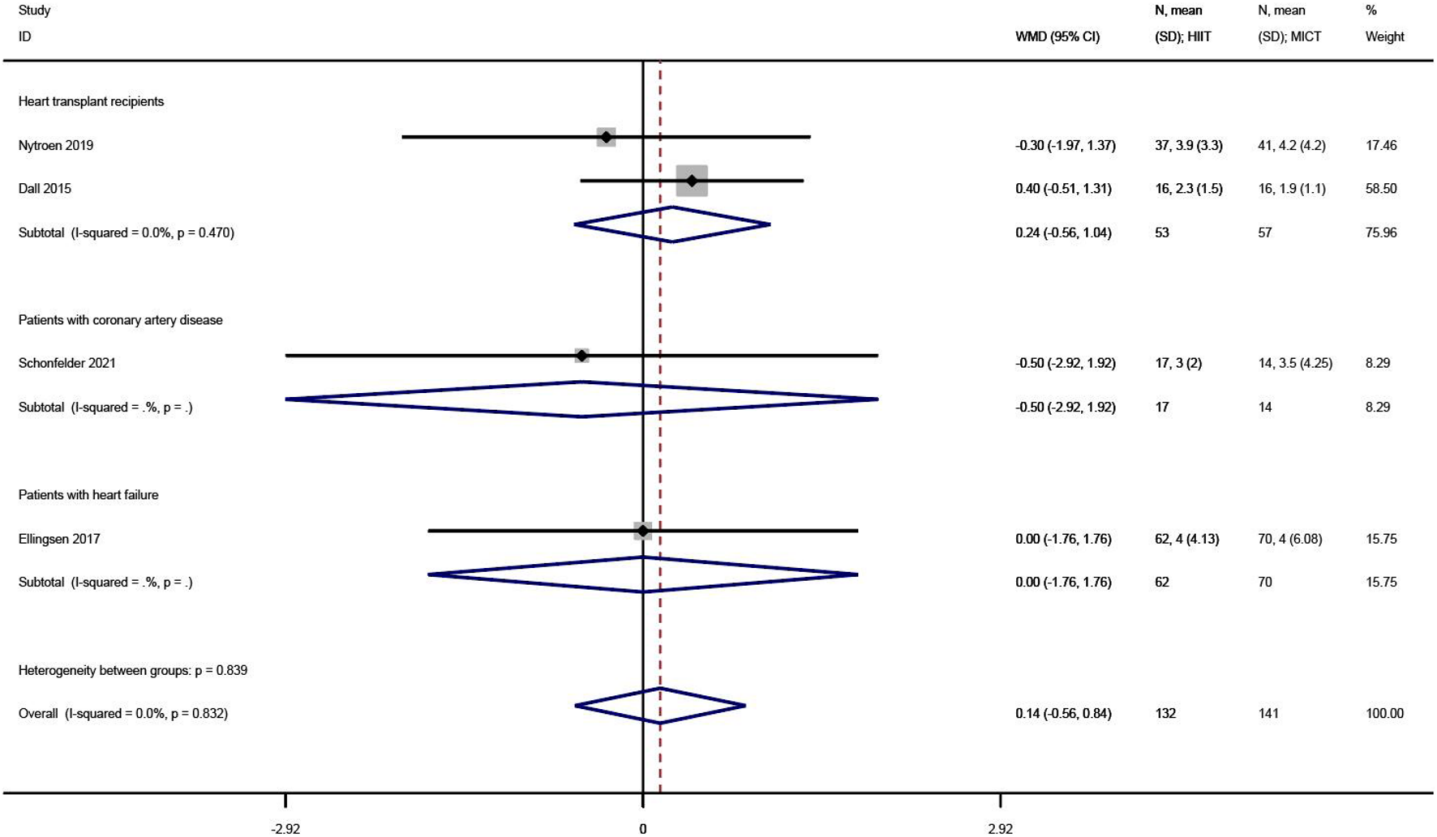
The results of meta-analyses of the effect of anxiety. The results of HIIT and MICT are shown as: mean, standard deviation (sample size).

Anxiety levels were reported in 4 of the 23 studies^27,30,36,38^. The WMD was used in the meta-analysis for effect size synthesis, and no between-study heterogeneity was observed (I^2^ = 0.0%, P = 0.832). The FE analysis showed that the difference in anxiety levels between the HIIT and MICT groups was not statistically significant (WMD = 0.14, 95% CI  − 0.56 ~ 0.84, Z = 0.39, P = 0.694), as shown in Fig. 7.

#### Additional analyses

**Figure 8:**
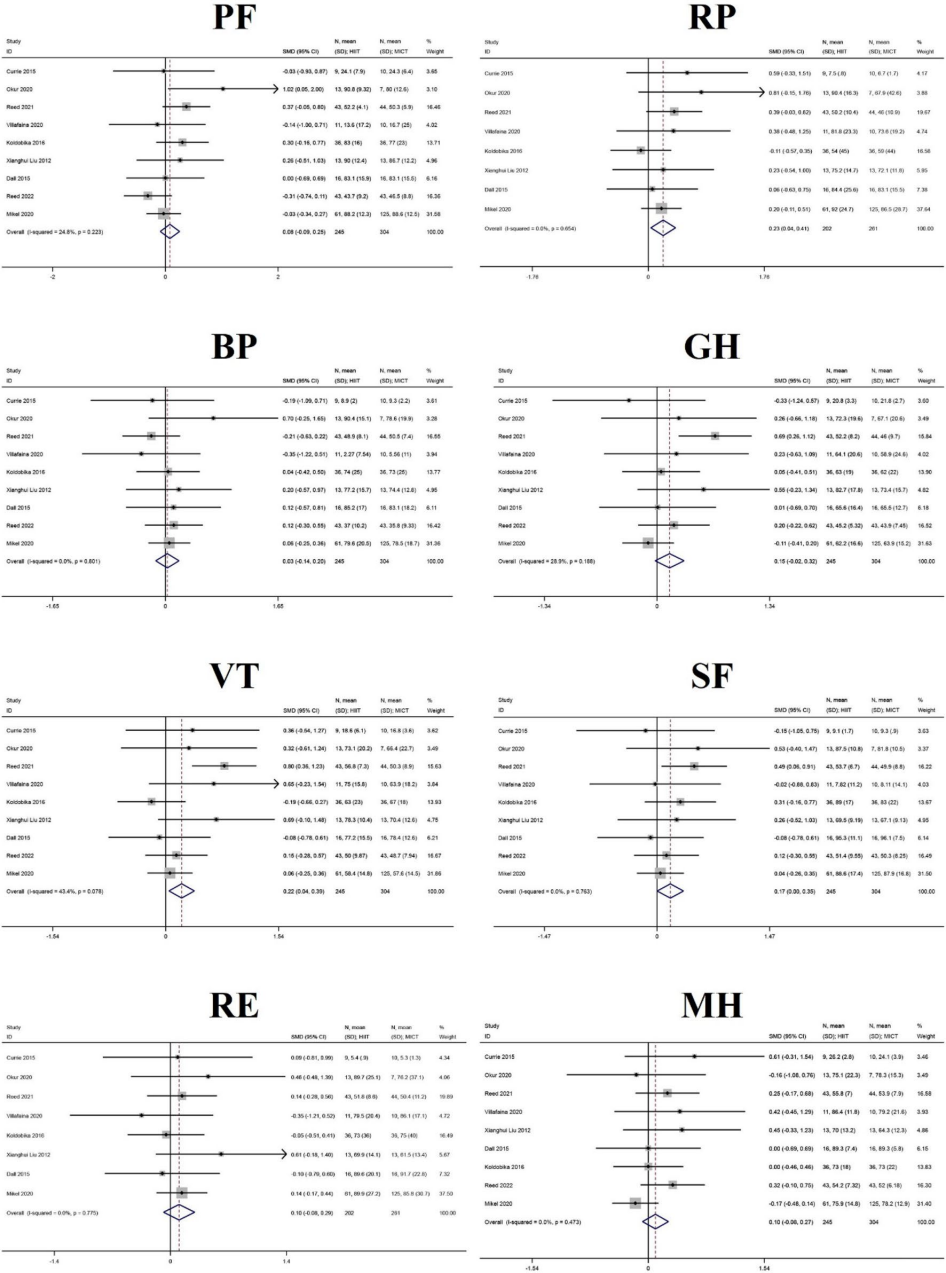
The results of meta-analyses of the effect of eight sub-dimensions in QOL. The results of HIIT and MICT are shown as: mean, standard deviation (sample size).

In addition to PCS and MCS, this study conducted additional analyses of sub-indicators for eight dimensions of quality of life: physical functioning (PF), role-physical (RP), bodily pain (BP), general health (GH), vitality (VT), social functioning (SF), role-emotional (RE), MH, and reported health transition (HT). Ten of the 23 studies reported, in detail, on the eight sub-dimensions of SF-36 and SF-12^25,28,29,32,33,35,36,41,47^. Therefore, additional analyses were performed on these dimensions. Surprisingly, the HIIT group was superior to the MICT group for three dimensions, namely RP, VT, and SF, and the differences were statistically significant. The results of the meta-analysis for RP (SMD = 0.23, 95% CI  0.04 ~ 0.41, Z = 2.36, P = 0.018) and the FE analysis of VT (SMD = 0.22, 95% CI  0.04 ~ 0.39, Z = 2.46, P = 0.014), and SF (SMD = 0.17, 95% CI  0.00 ~ 0.35, Z = 1.98, P = 0.048) are shown in Table 3 and Fig. 8, and further details are displayed in Supplement 3.

**Table 3:** The results of meta-analyses of the effect of eight dimensions of quality of life.

Dimension	Number of studies	Heterogeneity		Effect model	Meta analysis results		
		I2	P		SMD (95% CI)	Z	P
PF	9	24.80%	0.223	FE	0.08 (− 0.09,0.25)	0.91	0.362
RP	8	0.00%	0.654	FE	0.23 (0.04,0.41)	2.36	0.018
BP	9	0.00%	0.801	FE	0.03 (− 0.14,0.20)	0.33	0.743
GH	9	28.90%	0.188	FE	0.15 (− 0.02,0.32)	1.69	0.09
VT	9	43.40%	0.078	FE	0.22 (0.04,0.39)	2.46	0.014
SF	9	0.00%	0.763	FE	0.17 (0.00,0.35)	1.98	0.048
RE	8	0.00%	0.775	FE	0.10 (− 0.08,0.29)	1.09	0.276
MH	8	0.00%	0.473	FE	0.10 (− 0.08,0.27)	1.09	0.276

### Sensitivity analyses

**Figure 9:**
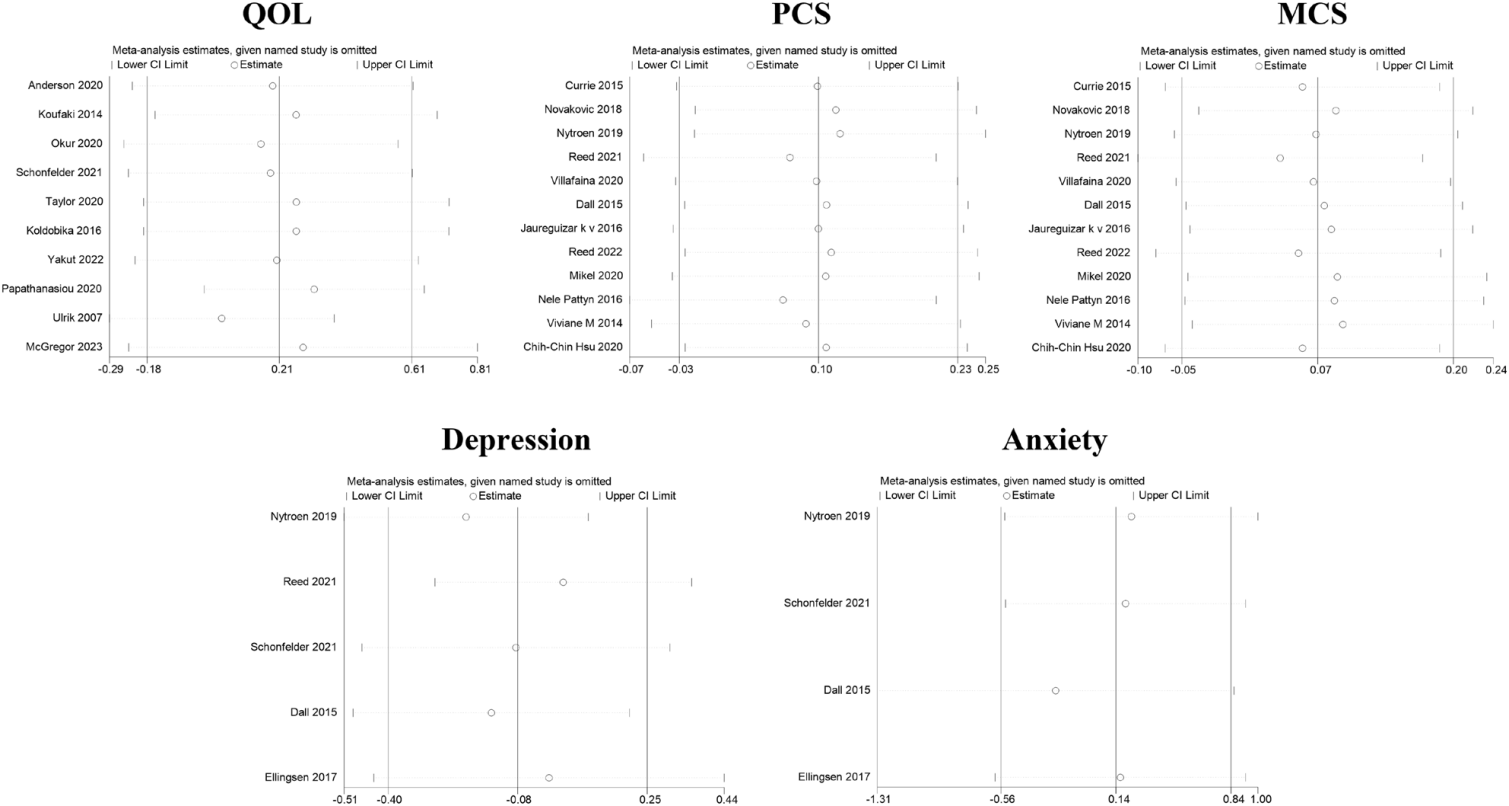
The results of sensitivity analyses.

In the sensitivity analyses for QOL, PCS, MCS, depression, and anxiety, after each of the included studies were excluded one-by-one, the overall effect values of the remaining studies fell within the 95% CI range of the original overall effect value, indicating that the meta-analysis results for QOL, PCS, MCS, depression, and anxiety were highly stable and contained no influential studies, as shown in Fig. 9.

### Publication bias

**Figure 10:**
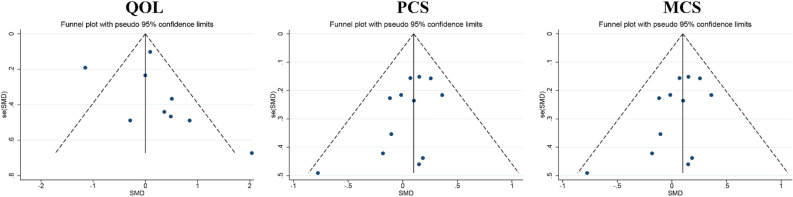
The results of publication bias.

Publication bias was assessed regarding QOL, PCS, and MCS. In the funnel plot of QOL, results were distributed on both sides of the symmetry axis. The funnel plots appeared to be asymmetric, suggesting that publication bias may exist in the data regarding QOL; however, the results of the egger test showed no publication bias (t = 1.21, P = 0.260). In the results both of PCS and MCS, the funnel plots were symmetric, suggesting that the data for PCS and MCS were not influenced by publication bias, as supported by the results of the egger test (t = − 1.89, P = 0.087 and t = 0.76, P = 0.466, respectively). According to the principle of inclusion and testing of publication bias, the analysis did not include depression and anxiety due to the limited availability of literature, with less than 10 studies available^48,49^, as shown in Fig. 10.

## Discussion

The current systematic review aimed to investigate the effects of HIIT and MICT on the QOL and MH of patients with CVDs by searching 8 electronic databases and evaluating 25 relevant articles. The study conducted a further analysis on the effects of HIIT and MICT on eight sub-dimensional indicators, including PF, RP, BP, GH, VT, SF, RE, and MH in QOL, with subgroup analyses based on population differences. The results showed that HIIT and MICT have similar effects on improving QOL and MH in patients with CVDs. However, HIIT is more conducive to the restoration of physiological functions and self-perceived energy, as well as to the alleviation of functional limitations associated with the condition, thus promoting the healthy development of the patient’s social adjustment. Notably, this effect was particularly pronounced in patients with CAD.

As the concept of health changes under the model of bio-psycho-social medicine, the measurement of health is gradually changing from a single physical health measurement to a multidimensional measurement of physical, psychological, social, and subjective well-being. The WHO define health as a state of complete physical, mental, and social well-being and not merely the absence of disease or infirmity^3^. QOL, as an important health assessment indicator, is defined as an individual’s perception of their position in life in the context of the culture and value systems in which they live and in relation to their goals, expectations, standards, and concerns. These include individual physiology, psychology, social function, and material state^50^. For patients with CVDs, QOL is a comprehensive concept centered on patients’ subjective feelings and encompassing multidimensional assessment indicators. Similar to cardiac function, blood markers, cardiovascular events, or other types of clinical indicators, QOL during a patient’s return visit is an important metric for evaluating the effectiveness of the health care services the patient received^6^. In contrast to traditional methods of survival analysis, the QOL evaluation system also includes an investigation of the patient’s physiological status. However, the difference is that the process is conducted from the patient’s subjective point of view, and therefore, the measurements can be more sensitive to the changes in physiological feedback, proprioception, and attitudes toward treatment during CR^35^. Therefore, health-related QOL is often measured using subjective questionnaires, including general health-related QOL (general tool) and health-related QOL in specific disease-related areas (specific tool)^51,52^. Currently, the majority of clinical trials involving CVDs include QOL as an essential indicator. Through the observation of QOL, physicians and caregivers can have a clearer understanding of the patient’s treatment progress from a multidimensional perspective and make timely adjustments to optimize CR.

Previous meta-analyses have demonstrated that HIIT is superior to MICT in improving physiological markers, such as flow mediated dilation, mitochondrial function in skeletal muscle, maximal oxygen consumption (VO_2max_), blood pressure, and lipid control in patients with CVDs^31,45,53^. However, the number of reports on subjective markers, such as QOL, is more limited. Both QOL and MH were included in this study as primary outcome indicators, and there were no strict inclusion criteria for participant eligibility, except for the type of disease. Therefore, general health-related QOL was used in the analysis of QOL, which has good reliability and validity^54^. Although there was little difference between HIIT and MICT regarding the improvement of PCS and MCS in the majority of patients, the effect of HIIT in improving PCS in patients with CAD was significant. This outcome, in part, supports the first hypothesis of the current study, which suggests that HIIT enhances the rehabilitative efficiency of physiological functions during CR in patients with CAD, which is consistent with the outcomes of two previous meta-analyses^13,55^. However, the difference to the present study is that other studies only included objective indexes, including VO_2max_, anaerobic threshold, and peak power output (PPO), whereas in the present study, PCS was evaluated on the basis of the patient’s proprioception. Therefore, it can be concluded that HIIT has a combined benefit for patients with CAD, which may be related to the physiological mechanisms by which HIIT positively affects several aspects of health. Regular HIIT promotes the onset of mitochondrial adaptations within skeletal muscle; an effect that not only enhances the efficiency of adenosine triphosphate supply^56^, but also contributes to the improvement of lipid oxidation and glucose metabolism, which leads to the regulation of blood pressure and blood lipids^57^. Concurrently, repetitive high- and low-intensity stimulation mediates myocardial remodeling, including an increase in ventricular wall thickness and ventricular volume. Myocardial adaptation improves the heart’s pumping capacity, thereby increasing cardiorespiratory fitness and aerobic endurance in exercisers^16,58^. Furthermore, the physiological adaptations induced by HIIT were also reflected in improvements to lactate threshold and exercise tolerance^59^. These factors may explain why HIIT induced superior improvements in RP, VT, and other sub-indices than those of MICT in the present study.

In addition, differences in PCS were related to different disease types, and the present study suggests the following explanations. This finding may be due to differences in pathological characteristics of patients with different types of CVDs, which leads to heterogeneity in the effectiveness of the training and feedback at the return visit. For example, in patients with tetralogy of Fallot, congenital ventricular septal defects lead to conditions such as pulmonary stenosis, overriding aorta, and right ventricular hypertrophy; thus, limiting the promotion of CRF by HIIT^60^. Tolerance and adaptation to exercise are also important factors, and heart transplant recipients may require longer cycles of CR to gradually adapt to high intensity training^61^. In contrast, the longest exercise cycle for the group of patients in the present study was only 36 weeks, and thus, good temporal efficacy may not have been achieved. In patients with ischemic heart disease, insufficient blood supply to the coronary arteries is prevalent^62^, which may affect the degree of saturation of the exercise, resulting in failure to meet the established intensity requirements and exercise goals. In addition, HIIT may cause arrhythmias in patients with atrial fibrillation (AF)^63^. In such cases, for safety reasons, physicians and caregivers may adjust the intensity of the exercise program. The experiments involving patients with AF in the present study used PPO as an index of intensity^47^. Unlike absolute indexes, such as maximum heart rate or VO_2max_, PPO is a relative index based on the patient’s own maximal power output as a baseline, and hence, may contribute to the differences in exercise intensity and intervention effects. Finally, in terms of the results reported for exercise intervention characteristics, despite the high intensity and difficulty of HIIT, adherence to both types of exercise exceeded 70%, with a difference of no more than 5%^64^. Concurrently, the duration of HIIT was generally shorter than that of MICT; therefore, HIIT improves the efficiency of CR.

MH is an important clinical assessment and includes the evaluation of many psychological factors. Among them, depression and anxiety are important indicators that often appear in self-assessment entries during the return visit of patients with clinical diseases^65,66^. Both diseases are closely related to patients’ QOL and social adjustment^67^. The presence of anxiety and depression can lead to psychological distress, dysfunction, and social problems, and in severe cases, can lead to heart disease, immune deficiency, and other physical health problems^68^. For patients with CVDs, a study conducted over a 10-year period found that the development of CVDs is strongly associated with poor mood. The prevalence of depression was 9.2% and 4.9% in patients with CAD and hypertension, respectively, while the prevalence of anxiety was 45.8% and 47.2% for patients with CAD and hypertension, respectively. Before percutaneous coronary intervention, anxiety was present in 70% of patients and definite depression in 38%^69^. Therefore, MH is particularly important in CR, and a favorable psychological state will have a positive effect on the individual’s efficiency in recovering from the disease, social adaptability, and psychological resilience. In recent years, MH disorders, including depression and anxiety, have been increasingly used as assessment indicators in the return visit of patients during rehabilitation. MH can be assessed primarily on the basis of scales such as the Beck Depression Inventory and Hospital Anxiety and Depression Scale^30,37^. Through the observation of the two, physicians and nursing staff can more intuitively understand the changes in the patients’ psychological state and negative emotions during CR, which is of certain significance for the development and adjustment of nursing care.

Currently, medication is the main method of intervention for depression and anxiety; however, adverse effects and addiction may lead to poor efficacy and affect the QOL of patients^70^. In recent years, with the increasing integration of physical medicine, exercise has been used as an MH intervention in selected clinical trials. There is evidence that elevated exercise intensity induces higher levels of MH improvement, e.g. HIIT may be superior to MICT in reducing depression and anxiety levels^27,71,72^. However, in this review, no significant differences were observed between the effects of HIIT and MICT on anxiety and depression among patients with CVDs. Therefore, the second hypothesis of this study cannot be accepted based on the current results. Notably, the sensitivity analyses of depression outcomes showed that depression data were unstable, which may be explained by the large heterogeneity between studies^27^. Nonetheless, the present study speculates that this result may still be due to differences in the duration and intensity of the intervention period.

In addition, the results of the QOL sub-indicator showed that HIIT improved SF better than did MICT. The present study suggests that this may be due to the different psychological mechanisms of the two exercise types in affecting SF. Some studies have found that high-intensity exercise is superior to other exercises in improving physical form, self-esteem, self-confidence, and positive mood^73^ and that acute HIIT may lead to greater exercise achievement and self-satisfaction^74^. Therefore, the present study suggests that appropriate exercise can improve MH, and HIIT may be more beneficial for enhancing social well-being, such as restoring confidence and returning to work, in patients with late CR.

Due to the unique physical condition of patients with CVDs, it is particularly important to prescribe the correct intensity and monitor the load of their physical exercise, considering the risk factors of inappropriate activity. By this means, a positive effect of recovery can be achieved only through reasonable exercise on the basis of avoiding the exacerbation of the patients’ condition^32^. Among the studies included in this paper, in addition to QOL as an outcome index, body mass index, heart rate, VO_2max_, 6 min walking performance, and exercise tolerance were among the additional variables measured to examine the effect of difference exercise regimens on patients.

In recent years, studies have confirmed that moderate HIIT can improve cardiopulmonary function and QOL in patients with CAD or HF. However, due to the alternating high- and low-intensity nature of the program, it is risky as a primary rehabilitation program for patients with myocardial infarction, tetralogy of Fallot, AF, and heart transplant recipients^28,30^. Therefore, this paper suggests that MICT performed with a constant load as the dominant training program, interspersed with HIIT, may have a positive impact on patients’ alleviation of negative emotions, restoration of exercise capacity, and promotion of overload recovery. Real-time monitoring of patients’ physiological load throughout training, for instance using cardio-pulmonary exercise testing, is beneficial for not only assessing the patient’s risk of cardiac emergencies, but also allowing the development of an individualized cardiac rehabilitation treatment strategy^29^. In addition, it is possible to record real-time heart rate, target heart rate, heart rate variability (HRV), blood glucose and lactate concentrations, VO_2max_, rating of perceived exertion, basal metabolic rate, and metabolic index, among other parameters, during exercise to give patients the best exercise experience with minimal invasiveness. This not only ensures activity and training quality, but also reduces cardiac burden and injury risk and prevents exacerbation and recurrence of the disease.

### Limitations and suggestions for future research

This study was conducted in strict accordance with the Preferred Reporting Items for Systematic Reviews and Meta-analysis (PRISMA) statement, but there are still some limitations: (i) there are some differences in intensity, duration, and frequency of intervention among studies, which produced large heterogeneity. Moreover, after sensitivity analysis and subgroup analysis, heterogeneity of some outcomes was not eliminated; (ii) some studies included small sample sizes, which may have influenced the results.

We recommend that future research should focus on expanding the following areas. First, questions regarding the extent to which internal control, exercise format, and sex differences influence the effects of HIIT need to be further explored. Nevertheless, due to a paucity of relevant literature and limited number of studies included in the present analysis, more high-quality studies need to be conducted to subsequently validate them. Second, the results of this study show that HIIT and MICT have similar effects on the improvement of QOL and MCS total scores in patients with CVDs, but HIIT has a significant advantage on the improvement of PCS total scores in patients with CAD. HIIT has a low time burden and high enjoyment levels. These factors improve compliance with HIIT, making it an effective alternative to MICT. It is recommended that HIIT be promoted as a form of exercise in the rehabilitation of patients with CAD. In this way, more rehabilitation options are available to patients with CVDs. Third, studies have shown that exercise preference and level of enjoyment affect exercise adherence^75^. Exercisers can choose exercise based on their personal preferences to improve exercise adherence. HIIT has similar beneficial effects on QOL and MH compared to MICT, which helps exercisers choose exercise patterns based on their preferences. HIIT has similar or higher levels of enjoyment and adherence than MICT^76^. Also, HIIT has a shorter workout duration than MICT^28^. Lack of time is one of the most common reasons for not exercising. Therefore, exercise forms with the least time investment and the same exercise effect may be an effective way to improve exercise adherence in the population^77,78^. Studies have shown that reducing the time needed to achieve health improvements can help increase adherence rates^79,80^. In this review, the efficacy of HIIT was superior to MICT. In addition, the vast majority of the 25 papers included in this study were found to include warm-up and cool-down periods in their exercise programs, indicating the importance of pre- exercise and post-exercise preparation. Fourth, the results of this study differ from the findings of Gomes et al. and Xiong et al.^81,82^, which may be due to differences in the pathological characteristics of patients with CVDs included in the present study. Future research should be targeted to a particular patient population, and the best exercise modality should be selected based on the pathological characteristics of the patient. Thus, it is recommended that future studies should refine the study population to obtain more precise outcomes.

## Conclusions

Three conclusions can be derived from this work: (1) The effects of HIIT and MICT on QOL were similar in patients with CVDs, but the physiologic health-promoting effects of HIIT were more pronounced in patients with CAD; (2) HIIT is more conducive to alleviating functional limitations based on physical problems, restoring self-perceived energy, and improving social adaptability; and (3) HIIT and MICT have similar moderating effects on MH, but HIIT increases the efficiency of exercise. Therefore, this study concluded that HIIT is highly valuable and should be promoted in the treatment and rehabilitation phase of patients with CVDs.

## Methods

This systematic review was registered at PROSPERO (CRD42022313051), followed the Cochrane guidelines, and complied with the PRISMA checklist^48,49^.

### Data sources and search strategy

Eight databases were searched including Web of Science (Science and Social Science Citation Index), MEDLINE(R)ALL, Embase, Cochrane Central Register of Controlled Trials (CENTRAL), CINAHL, China National Knowledge Infrastructure, Wanfang Database, and China Science and Technology Journal Database. Using PubMed as an example, see Box 1 for details of the search terms. The search was conducted from the database establishment to July 2023. The relevant information was sought through the included references, and unpublished academic literature was not searched.

Box 1 PubMed search strategy #1 ((HIIT[Title/Abstract]) OR (SIT[Title/Abstract]) OR (AIC[Title/Abstract]) OR (High intensity interval training[MeSH Terms]) OR (High intensity interval training[Title/Abstract]) OR (High intensity functional training[Title/Abstract]) OR (High intensity power training[Title/Abstract]) OR (High intensity endurance training[Title/Abstract]) OR (High intensity circuit training[Title/Abstract]) OR (Sprint interval training[Title/Abstract]) OR (Aerobic interval training[Title/Abstract]) OR (Interval training[Title/Abstract])).

#2 ((AE[Title/Abstract]) OR (AT[Title/Abstract]) OR (MICT[Title/Abstract]) OR (ACT[MeSH Terms]) OR (Aerobic exercise[MeSH Terms]) OR (Moderate intensity continuous training[Title/Abstract]) OR (Aerobic continuous training[MeSH Terms]) OR (Moderate intensity training[Title/Abstract]) OR (Continuous training[Title/Abstract])).

#3 ((Cardiovascular disease[MeSH Terms]) OR (Coronary heart disease[MeSH Terms]) OR (Angina pectoris[MeSH Terms]) OR (Hypertension[MeSH Terms]) OR (Hyperlipidemias[MeSH Terms]) OR (Heart failure[MeSH Terms]) OR (Myocardial infarction[MeSH Terms]) OR (Heart transplantation[MeSH Terms]) OR (Stroke[MeSH Terms]) OR (Arrhythmias, Cardiac[MeSH Terms]) OR (Atherosclerosis[MeSH Terms]) OR (Pericarditis[MeSH Terms]) OR (Myocarditis[MeSH Terms]) OR (Cardiomyopathies[MeSH Terms]) OR (Heart defects, Congenital[MeSH Terms]) OR (Heart valve diseases[MeSH Terms]) OR (Tetralogy of Fallot[MeSH Terms]) OR (Heart transplantation[MeSH Terms]) OR (Aneurysm[MeSH Terms]) OR (Cardiac conduction system disease[MeSH Terms]) OR (Endocarditis[MeSH Terms]) OR (Thromboembolism[MeSH Terms]) OR (Thrombosis[MeSH Terms]) OR (Aortic valve stenosis[MeSH Terms]) OR (Aortic valve closure insufficiency[MeSH Terms]) OR (Atrial fibrillation[MeSH Terms]) OR (Pericardial effusion[MeSH Terms]) OR (Hypertrophy, Left ventricular[MeSH Terms]) OR (Hypertrophy, Right ventricular[MeSH Terms]) OR (Cardiomyopathy, Hypertrophic, Familial[MeSH Terms]) OR (Mitral valve prolapse[MeSH Terms]) OR (Chronic venous insufficiency[Title/Abstract]) OR (Elastic plaque[Title/Abstract]) OR (Abnormal heart structure[Title/Abstract]) OR (Arteriostenosis[Title/Abstract]) OR (Arterial stenosis[Title/Abstract])).

#4 ((QOL[Title/Abstract]) OR (MH[Title/Abstract]) OR (Life quality[Title/Abstract]) OR (Health related quality of life[Title/Abstract]) OR (Quality of life[MeSH Terms]) OR (Mental health[MeSH Terms]) OR (Mental health[MeSH Terms]) OR (Mental well-being[Title/Abstract])).

#5 #1 AND #2 AND #3 AND #4

### Study selection

Based on the PICOS, the relevant studies were selected (Table 4). The inclusion criteria were randomized controlled trials (RCTs); literature written in English or Chinese and published in academic journals; studies involving patients with all currently known types of CVDs; comparison between HIIT and MICT exercise interventions; the form of exercise must be consistent with the characteristics of HIIT and MICT; the results were discussed based on QOL and MH, including depression and anxiety, of patients with CVDs. Literature with combined exercise or did not group exercise was excluded. The outcome indicators were QOL and MH, including depression and anxiety, based on subjective scales.

**Table 4:** PICOS-based eligibility criteria.

PICOS	Criteria
Participants	Patients with cardiovascular disease
Intervention	HIIT or MICT
Comparison	HIIT or MICT in patients with cardiovascular disease
Outcome	Quality of life (QOL evaluation scale) and mental health (depression and anxiety evaluation scale)
Study design	Randomized controlled trial

### Data extraction

According to the screening criteria, two researchers independently read the title and abstract of the retrieved literature. Then, the full texts of studies that met the primary screening criteria were screened and the relevant literature was obtained. Disagreements regarding the literature selection were resolved through discussions among the researchers.

Literature screening was performed using Endnote X9 software by two independent investigators; first, with a round of screening based on title and abstract, and second, by downloading the full text of the literature to determine its eligibility for inclusion. Extracted data included the year of publication, country, patient details, number of cases, age, exercise intervention, and outcome indicators.

### Quality assessment

The quality of RCTs was evaluated using the tools of the Cochrane Collaboration^83^. Grading of Recommendations and Assessment Development and Evaluation (GRADE) was used to evaluate the quality of evidence with a web-based version (https://gradepro.org). In this study, based on the GRADE criteria for grading the quality of evidence, the criteria for assessing and downgrading the quality of literature were categorized into five items: risk of bias, inconsistency, indirectness, imprecision, and other considerations, according to which the risk level was evaluated^84^. Any differences in the quality assessment were resolved through discussions among researchers, and if consensus could not be reached, senior researchers were consulted.

### Data analysis

Meta-analyses were performed using Stata 17.0 software. All data extracted in this study were continuous variables. If the evaluation measures of outcome indicators were the same, the weighted mean difference (WMD) was used for effect size comparison; if the evaluation measures of outcome indicators were different, the standardized mean difference (SMD) was used for effect size comparison. The calculation of WMD/SMD and the analysis of the results in this study were performed using the methodology described by Khan and Schober^85,86^. If a trial included multiple exercise interventions and a shared control group, we combined the interventions according to the appropriate formula in the Cochrane Systematic Review of Interventions Manual^48^. The I_2_ test was used to assess heterogeneity, with an I_2_ of 25–50% classified as low heterogeneity, 50–75% as moderate heterogeneity, and > 75% as severe heterogeneity^87^. The FE model was used when I_2_ ≤ 50% and the RE model was used when I_2_ > 50%. The results of the meta-analysis were presented in the form of forest maps. Literature publication bias was assessed using funnel plots and the Egger’s test^88^. A sensitivity analysis was performed by using the one-by-one elimination method.

### Supplementary Information

Supplementary Information 1.

Supplementary Information 2.

Supplementary Information 3.

Supplementary Information 4.

Supplementary Information 5.

## Data Availability

Data are available upon reasonable request. The datasets used and/or analyzed during the current study are available from the corresponding author upon reasonable request.

## References

[1] GDAI Collaborators (2020). Global burden of 369 diseases and injuries in 204 countries and territories, 1990–2019. Lancet.

[2] Vyas A, Desai R, Patel V (2023). Rising burden of cardiovascular disease risk factors and acute cardiac events in young adults with comorbid depression: A comparison nationwide US cohorts hospitalized 10-years apart. Curr. Probl. Cardiol..

[3] Organization WH (1946). Constitution of the world health organization. Am. J. Public Health Nations Health.

[4] Vahedi S (2010). World health organization quality-of-life scale (WHOQOL-BREF): Analyses of their item response theory properties based on the graded responses model. Iran. J. Psychiatry.

[5] Weberg M, Hjermstad MJ, Hilmarsen CW, Oldervoll L (2013). Inpatient cardiac rehabilitation and changes in self-reported health related quality of life–a pilot study. Ann. Phys. Rehabil. Med..

[6] Höfer S (2012). The MacNew heart disease health-related quality of life questionnaire in patients with angina and patients with ischemic heart failure. Value Health J. Int. Soc. Pharmacoecon. Outcomes Res..

[7] Heran BS (2011). Exercise-based cardiac rehabilitation for coronary heart disease. Cochrane Database Syst. Rev..

[8] Pelliccia A (2021). 2020 ESC guidelines on sports cardiology and exercise in patients with cardiovascular disease. Eur. Heart J..

[9] Bruseghini P (2020). High intensity interval training does not have compensatory effects on physical activity levels in older adults. Int. J. Environ. Res. Public Health.

[10] Khoury R, Nagy C (2023). Running from stress: A perspective on the potential benefits of exercise-induced small extracellular vesicles for individuals with major depressive disorder. Front. Mol. Biosci..

[11] Arnett DK (2019). 2019 ACC/AHA guideline on the primary prevention of cardiovascular disease: A report of the American College of Cardiology/American Heart Association task force on clinical practice guidelines. Circulation.

[12] Novakovic M (2018). Exercise training in adults with repaired tetralogy of Fallot: A randomized controlled pilot study of continuous versus interval training. Int. J. Cardiol..

[13] Du L (2021). Effect of high-intensity interval training on physical health in coronary artery disease patients: A meta-analysis of randomized controlled trials. J. Cardiovasc. Dev. Dis..

[14] Garber CE (2011). American college of sports medicine position stand. Quantity and quality of exercise for developing and maintaining cardiorespiratory, musculoskeletal, and neuromotor fitness in apparently healthy adults: Guidance for prescribing exercise. Med. Sci. Sports Exerc..

[15] Balady GJ (2007). Core components of cardiac rehabilitation/secondary prevention programs: 2007 update: A scientific statement from the American Heart Association Exercise, Cardiac Rehabilitation, and Prevention Committee, the Council on Clinical Cardiology; the Councils on Cardiovascular Nursing, Epidemiology and Prevention, and Nutrition, Physical Activity, and Metabolism; and the American Association of Cardiovascular and Pulmonary Rehabilitation. Circulation.

[16] Edwards JJ, Griffiths M, Deenmamode AHP, O’Driscoll JM (2023). High-intensity interval training and cardiometabolic health in the general population: A systematic review and meta-analysis of randomised controlled trials. Sports Med..

[17] Twerenbold S (2023). Short-term high-intensity interval training improves micro- but not macrovascular function in hypertensive patients. Scand. J. Med. Sci. Sports.

[18] Gevaert AB (2023). Effect of training on vascular function and repair in heart failure with preserved ejection fraction. JACC Heart Fail..

[19] Qin Y, Kumar Bundhun P, Yuan ZL, Chen MH (2022). The effect of high-intensity interval training on exercise capacity in post-myocardial infarction patients: A systematic review and meta-analysis. Eur. J. Prev. Cardiol..

[20] de Souza Mesquita FO (2023). Effect of high-intensity interval training on exercise capacity, blood pressure, and autonomic responses in patients with hypertension: A systematic review and meta-analysis. Sports Health.

[21] MacInnis MJ, Gibala MJ (2017). Physiological adaptations to interval training and the role of exercise intensity. J. Physiol..

[22] Blasco-Peris C (2022). Effects of exergaming in patients with cardiovascular disease compared to conventional cardiac rehabilitation: A systematic review and meta-analysis. Int. J. Environ. Res. Public Health.

[23] Wang C (2022). The effects of high-intensity interval training on exercise capacity and prognosis in heart failure and coronary artery disease: A systematic review and meta-analysis. Cardiovasc. Ther..

[24] Donelli da Silveira A (2020). High-intensity interval training is effective and superior to moderate continuous training in patients with heart failure with preserved ejection fraction: A randomized clinical trial. Eur. J. Prev. Cardiol..

[25] Currie KD, Bailey KJ, Jung ME, McKelvie RS, MacDonald MJ (2015). Effects of resistance training combined with moderate-intensity endurance or low-volume high-intensity interval exercise on cardiovascular risk factors in patients with coronary artery disease. J. Sci. Med. Sport.

[26] Koufaki P, Mercer TH, George KP, Nolan J (2014). Low-volume high-intensity interval training vs continuous aerobic cycling in patients with chronic heart failure: A pragmatic randomised clinical trial of feasibility and effectiveness. J. Rehabil. Med..

[27] Nytroen K (2019). Effect of high-intensity interval training in de novo heart transplant recipients in scandinavia. Circulation.

[28] Okur I, Aksoy CC, Yaman F, Sen T (2022). Which high-intensity interval training program is more effective in patients with coronary artery disease?. Int. J. Rehabil. Res..

[29] Reed JL (2022). The effects of high-intensity interval training, Nordic walking and moderate-to-vigorous intensity continuous training on functional capacity, depression and quality of life in patients with coronary artery disease enrolled in cardiac rehabilitation: A randomized controlled trial (CRX study). Progress Cardiovasc. Dis..

[30] Schonfelder M (2021). Effect of different endurance training protocols during cardiac rehabilitation on quality of life. Am. J. Med..

[31] Taylor JL (2020). Short-term and long-term feasibility, safety, and efficacy of high-intensity interval training in cardiac rehabilitation: The FITR heart study randomized clinical trial. JAMA Cardiol..

[32] Villafaina S, Giménez-Guervós Pérez M, Fuentes-García JP (2020). Comparative effects of high-intensity interval training vs moderate-intensity continuous training in phase iii of a tennis-based cardiac rehabilitation program: A pilot randomized controlled trial. Sustainability.

[33] Koldobika VJ (2016). Effect of high-intensity interval versus continuous exercise training on functional capacity and quality of life in patients with coronary artery disease. J. Cardiopulm. Rehabil. Prev..

[34] Yakut H (2022). Effect of home-based high-intensity interval training versus moderate-intensity continuous training in patients with myocardial infarction: A randomized controlled trial. Irish J. Med. Sci..

[35] Liu XH, Hao XM (2012). Effects of interval exercise on quality of life and plasma CRAMP in hypertension patients. J. Xi’an Phys. Educ. Univ..

[36] Dall CH (2015). Effect of moderate- versus high-intensity exercise on vascular function, biomarkers and quality of life in heart transplant recipients: A randomized, crossover trial. J. Heart Lung Transplant..

[37] Hsu CC, Fu TC, Huang SC, Chen CP, Wang JS (2021). Increased serum brain-derived neurotrophic factor with high-intensity interval training in stroke patients: A randomized controlled trial. Ann. Phys. Rehabil. Med..

[38] Ellingsen Ø (2017). High-intensity interval training in patients with heart failure with reduced ejection fraction. Circulation.

[39] Dunford EC (2021). Brief vigorous stair climbing effectively improves cardiorespiratory fitness in patients with coronary artery disease: A randomized trial. Front. Sports Act. Living.

[40] McGregor G (2023). High-intensity interval training in cardiac rehabilitation: A multi-centre randomised controlled trial. Eur. J. Prev. Cardiol..

[41] Tous-Espelosín M, Gorostegi-Anduaga I, Corres P, MartinezAguirre-Betolaza A, Maldonado-Martín S (2020). Impact on health-related quality of life after different aerobic exercise programs in physically inactive adults with overweight/obesity and primary hypertension: Data from the EXERDIET-HTA study. Int. J. Environ. Res. Public Health.

[42] Pattyn N (2016). The long-term effects of a randomized trial comparing aerobic interval versus continuous training in coronary artery disease patients: 1-year data from the SAINTEX-CAD study. Eur. J. Prev. Cardiol..

[43] Papathanasiou JV (2020). Group-based cardiac rehabilitation interventions. A challenge for physical and rehabilitation medicine physicians: A randomized controlled trial. Eur. J. Phys. Rehabil. Med..

[44] Moholdt TT (2009). Aerobic interval training versus continuous moderate exercise after coronary artery bypass surgery: A randomized study of cardiovascular effects and quality of life. Am. Heart J..

[45] Wisløff U (2007). Superior cardiovascular effect of aerobic interval training versus moderate continuous training in heart failure patients: A randomized study. Circulation.

[46] Conraads VM (2015). Aerobic interval training and continuous training equally improve aerobic exercise capacity in patients with coronary artery disease: The SAINTEX-CAD study. Int. J. Cardiol..

[47] Reed JL (2022). Effect of high-intensity interval training in patients with atrial fibrillation: A randomized clinical trial. JAMA Netw. Open.

[48] Higgins JPT (2019). Cochrane Handbook for Systematic Reviews of Interventions.

[49] Page MJ (2021). PRISMA 2020 explanation and elaboration: Updated guidance and exemplars for reporting systematic reviews. BMJ.

[50] WHO (1993). The Development of the WHO Quality of Life Assessment Instrument.

[51] Guyatt GH, Feeny DH, Patrick DL (1993). Measuring health-related quality of life. Ann. Intern. Med..

[52] Crosby RD, Kolotkin RL, Williams GR (2003). Defining clinically meaningful change in health-related quality of life. J. Clin. Epidemiol..

[53] Okamura M, Shimizu M, Yamamoto S, Nishie K, Konishi M (2023). High-intensity interval training versus moderate-intensity continuous training in patients with heart failure: A systematic review and meta-analysis. Heart Fail. Rev..

[54] Mchoney CA, Ware JE, Lu JFR (1994). The medical out- comes study( MOS) 36-item short form health survey( SF- 36) III. Tests of data quality, scaling assumptions and reliability across diverse patient groups. Med. Care.

[55] Hannan AL (2018). High-intensity interval training versus moderate-intensity continuous training within cardiac rehabilitation: A systematic review and meta-analysis. Open Access J. Sports Med..

[56] Granata C (2021). High-intensity training induces non-stoichiometric changes in the mitochondrial proteome of human skeletal muscle without reorganisation of respiratory chain content. Nat. Commun..

[57] Lu Y, Wiltshire HD, Baker JS, Wang Q, Ying S (2023). The effect of Tabata-style functional high-intensity interval training on cardiometabolic health and physical activity in female university students. Front. Physiol..

[58] Engel LE (2022). The high-intensity interval training mitigates the cardiac remodeling in spontaneously hypertensive rats. Life Sci..

[59] Zaenker P (2018). High-intensity interval training combined with resistance training improves physiological capacities, strength and quality of life in multiple sclerosis patients: A pilot study. Eur. J. Phys. Rehabil. Med..

[60] Pelosi C (2023). Daily life and psychosocial functioning of adults with congenital heart disease: A 40–53 years after surgery follow-up study. Clin. Res. Cardiol. Off. J. Ger. Card. Soc..

[61] Rafique M (2023). Effects of high-intensity interval training on cardiac remodelling, function and coronary microcirculation in de novo heart transplant patients: a substudy of the HITTS randomised controlled trial. BMJ Open Sport Exerc. Med..

[62] Querio G (2021). Ischemic heart disease and cardioprotection: Focus on estrogenic hormonal setting and microvascular health. Vasc. Pharmacol..

[63] Marcus GM (2022). Individualized studies of triggers of paroxysmal atrial fibrillation: The I-STOP-AFib randomized clinical trial. JAMA Cardiol..

[64] Bartlett JD (2011). High-intensity interval running is perceived to be more enjoyable than moderate-intensity continuous exercise: Implications for exercise adherence. J. Sports Sci..

[65] Scott KM (2007). Depression-anxiety relationships with chronic physical conditions: Results from the World Mental Health Surveys. J. Affect. Disord..

[66] Minhas S (2022). Mind-body connection: Cardiovascular sequelae of psychiatric illness. Curr. Probl. Cardiol..

[67] Jacobson NC, Newman MG (2017). Anxiety and depression as bidirectional risk factors for one another: A meta-analysis of longitudinal studies. Psychol. Bull..

[68] Roest AM, Martens EJ, de Jonge P, Denollet J (2010). Anxiety and risk of incident coronary heart disease: A meta-analysis. J. Am. Coll. Cardiol..

[69] Roth GA, Mensah GA, Johnson CO (2020). Global burden of cardiovascular diseases and risk factors, 1990–2019: Update from the GBD 2019 study. J. Am. Coll. Cardiol..

[70] Almohammed OA (2022). Antidepressants and health-related quality of life (HRQoL) for patients with depression: Analysis of the medical expenditure panel survey from the United States. PloS One.

[71] Martland R, Mondelli V, Gaughran F, Stubbs B (2020). Can high-intensity interval training improve physical and mental health outcomes? A meta-review of 33 systematic reviews across the lifespan. J. Sports Sci..

[72] Martland R (2022). Can high-intensity interval training improve mental health outcomes in the general population and those with physical illnesses? A systematic review and meta-analysis. Br. J. Sports Med..

[73] Shang Y, Xie HD, Yang SY (2021). The relationship between physical exercise and subjective well-being in college students: The mediating effect of body image and self-esteem. Front. Psychol..

[74] Bourke M (2022). The acute affective response to physical activity in people with depression: A meta-analysis. J. Affect. Disord..

[75] Keogh JW, Grigg J, Vertullo CJ (2018). Is high-intensity interval cycling feasible and more beneficial than continuous cycling for knee osteoarthritic patients? Results of a randomised control feasibility trial. PeerJ.

[76] Vella CA, Taylor K, Drummer D (2017). High-intensity interval and moderate-intensity continuous training elicit similar enjoyment and adherence levels in overweight and obese adults. Eur. J. Sport Sci..

[77] Ana K, Barbara F, Emily P, Jennifer JH (2019). The effects of aerobic exercise intensity on memory in older adults. Appl. Physiol. Nutr. Metab..

[78] Smith-Ryan AE, Trexler ET, Wingfield HL, Blue MN (2016). Effects of high-intensity interval training on cardiometabolic risk factors in overweight/obese women. J. Sports Sci..

[79] Heinrich KM, Patel PM, O’Neal JL, Heinrich BS (2014). High-intensity compared to moderate-intensity training for exercise initiation, enjoyment, adherence, and intentions: An intervention study. BMC Public Health.

[80] Ito S (2019). High-intensity interval training for health benefits and care of cardiac diseases—The key to an efficient exercise protocol. World J. Cardiol..

[81] Gomes VA (2023). Comparison of high-intensity interval training to moderate-intensity continuous training for functioning and quality of life in survivors of COVID-19 (COVIDEX): Protocol for a randomized controlled trial. Phys. Ther..

[82] Xiong X (2022). Which type of exercise during radiation therapy is optimal to improve fatigue and quality of life in men with prostate cancer? A Bayesian network analysis. Eur. Urol. Open Sci..

[83] Higgins JP (2011). The cochrane collaboration’s tool for assessing risk of bias in randomised trials. BMJ.

[84] Kumar A, Miladinovic B, Guyatt GH, Schünemann HJ, Djulbegovic B (2016). GRADE guidelines system is reproducible when instructions are clearly operationalized even among the guidelines panel members with limited experience with GRADE. J. Clin. Epidemiol..

[85] Schober P, Mascha EJ, Vetter TR (2021). Statistics from A (Agreement) to Z (Z score): A guide to interpreting common measures of association, agreement, diagnostic accuracy, effect size, heterogeneity, and reliability in medical research. Anesth. Analg..

[86] Khan S (2020). Meta-Analysis: Methods for Health and Experimental Studies.

[87] Jeffery M (2009). Interval training versus continuous training in patients with chronic obstructive pulmonary disease. J. Cardiopulm. Rehabil. Prev..

[88] Egger M, Davey Smith G, Schneider M, Minder C (1997). Bias in meta-analysis detected by a simple, graphical test. BMJ.

