# Supplementary material for: High-intensity interval training versus moderate-intensity continuous training on patient quality of life in cardiovascular disease: a systematic review and meta-analysis
Source: Sci Rep. 2023 Aug 25;13:13915. doi: 10.1038/s41598-023-40589-5 (PMC10457360; doi:10.1038/s41598-023-40589-5)
Supplement: Supplementary file 1 — Supplementary Information 1. [file 41598_2023_40589_MOESM1_ESM.docx]

#1 ((HIIT[Title/Abstract]) OR (SIT[Title/Abstract]) OR (AIC[Title/Abstract]) OR (High intensity interval training[MeSH Terms]) OR (High intensity interval training[Title/Abstract]) OR (High intensity functional training[Title/Abstract]) OR (High intensity power training[Title/Abstract]) OR (High intensity endurance training[Title/Abstract]) OR (High intensity circuit training[Title/Abstract]) OR (Sprint interval training[Title/Abstract]) OR (Aerobic interval training[Title/Abstract]) OR (Interval training[Title/Abstract]))

#2 ((AE[Title/Abstract]) OR (AT[Title/Abstract]) OR (MICT[Title/Abstract]) OR (ACT[MeSH Terms]) OR (Aerobic exercise[MeSH Terms]) OR (Moderate intensity continuous training[Title/Abstract]) OR (Aerobic continuous training[MeSH Terms]) OR (Moderate intensity training[Title/Abstract]) OR (Continuous training[Title/Abstract]))

#3 ((Cardiovascular disease[MeSH Terms]) OR (Coronary heart disease[MeSH Terms]) OR (Angina pectoris[MeSH Terms]) OR (Hypertension[MeSH Terms]) OR (Hyperlipidemias[MeSH Terms]) OR (Heart failure[MeSH Terms]) OR (Myocardial infarction[MeSH Terms]) OR (Heart transplantation[MeSH Terms]) OR (Stroke[MeSH Terms]) OR (Arrhythmias, Cardiac[MeSH Terms]) OR (Atherosclerosis[MeSH Terms]) OR (Pericarditis[MeSH Terms]) OR (Myocarditis[MeSH Terms]) OR (Cardiomyopathies[MeSH Terms]) OR (Heart defects, Congenital[MeSH Terms]) OR (Heart valve diseases[MeSH Terms]) OR (Tetralogy of fallot[MeSH Terms]) OR (Heart transplantation[MeSH Terms]) OR (Aneurysm[MeSH Terms]) OR (Cardiac conduction system disease[MeSH Terms]) OR (Endocarditis[MeSH Terms]) OR (Thromboembolism[MeSH Terms]) OR (Thrombosis[MeSH Terms]) OR (Aortic valve stenosis[MeSH Terms]) OR (Aortic valve closure insufficiency[MeSH Terms]) OR (Atrial fibrillation[MeSH Terms]) OR (Pericardial effusion[MeSH Terms]) OR (Hypertrophy, Left ventricular[MeSH Terms]) OR (Hypertrophy, Right ventricular[MeSH Terms]) OR (Cardiomyopathy, Hypertrophic, Familial[MeSH Terms]) OR (Mitral valve prolapse[MeSH Terms]) OR (Chronic venous insufficiency[Title/Abstract]) OR (Elastic plaque[Title/Abstract]) OR (Abnormal heart structure[Title/Abstract]) OR (Arteriostenosis[Title/Abstract]) OR (Arterial stenosis[Title/Abstract]))

#4 ((QOL[Title/Abstract]) OR (MH[Title/Abstract]) OR (Life quality[Title/Abstract]) OR (Health related quality of life[Title/Abstract]) OR (Quality of life[MeSH Terms]) OR (Mental health[MeSH Terms]) OR (Mental health[MeSH Terms]) OR (Mental well-being[Title/Abstract]))

#5 #1 AND #2 AND #3 AND #4

Box 1 PubMed search strategy
