# Supplementary material for: High-intensity interval training versus moderate-intensity continuous training on patient quality of life in cardiovascular disease: a systematic review and meta-analysis
Source: Sci Rep. 2023 Aug 25;13:13915. doi: 10.1038/s41598-023-40589-5 (PMC10457360; doi:10.1038/s41598-023-40589-5)
Supplement: Supplementary file 3 — Supplementary Information 3. [file 41598_2023_40589_MOESM3_ESM.docx]

**Author(s):**

**Question:** HIIT compared to MICT for patients with cardiovascular disease

**Setting:**

**Bibliography:**

| **Certainty assessment** | | | | | | | **No of patients** | | **Effect** | | **Certainty** | **Importance** |
| --- | --- | --- | --- | --- | --- | --- | --- | --- | --- | --- | --- | --- |
| **No of studies** | **Study design** | **Risk of bias** | **Inconsistency** | **Indirectness** | **Imprecision** | **Other considerations** | **HIIT** | **MICT** | **Relative (95% CI)** | **Absolute (95% CI)** |  |  |
| **QOL** | | | | | | | | | | | | |
| 10 | randomised trials | not serious | serious^a^ | not serious | not serious | none | 385 | 388 | - | SMD **0.21 SD higher** (0.18 lower to 0.61 higher) | ⨁⨁⨁◯ Moderate |  |
| **PCS** | | | | | | | | | | | | |
| 12 | randomised trials | not serious | serious^a^ | not serious | serious | none | 440 | 519 | - | SMD **0.10 SD higher** (0.03 lower to 0.23 higher) | ⨁⨁⨁◯ Moderate |  |
| **MCS** | | | | | | | | | | | | |
| 12 | randomised trials | not serious | serious^a^ | not serious | serious | none | 440 | 519 | - | SMD **0.07 SD higher** (0.05 lower to 0.20 higher) | ⨁⨁⨁◯ Moderate |  |
| **Depress** | | | | | | | | | | | | |
| 5 | randomised trials | not serious | serious^a^ | not serious | serious^b,c^ | none | 175 | 185 | - | SMD **0.08 SD lower** (0.40 lower to 0.25 higher) | ⨁⨁◯◯ Low |  |
| **Anxiety** | | | | | | | | | | | | |
| 4 | randomised trials | not serious | not serious | not serious | serious^c^ | none | 132 | 141 | - | MD **0.14 higher** (0.56 lower to 0.84 higher) | ⨁⨁⨁◯ Moderate |  |

**CI:** confidence interval; **MD:** mean difference; **SMD:** standardised mean difference

**Explanations:**

a. The span of the research population is large.

b. 25%< I2<75%

c. total sample size is less than optimal information size
