# Supplementary material for: High-intensity interval training versus moderate-intensity continuous training on patient quality of life in cardiovascular disease: a systematic review and meta-analysis
Source: Sci Rep. 2023 Aug 25;13:13915. doi: 10.1038/s41598-023-40589-5 (PMC10457360; doi:10.1038/s41598-023-40589-5)
Supplement: Supplementary file 4 — Supplementary Information 4. [file 41598_2023_40589_MOESM4_ESM.docx]

**Supplemental 3 The results of meta-analyses of the effect of eight dimensions in QOL.**


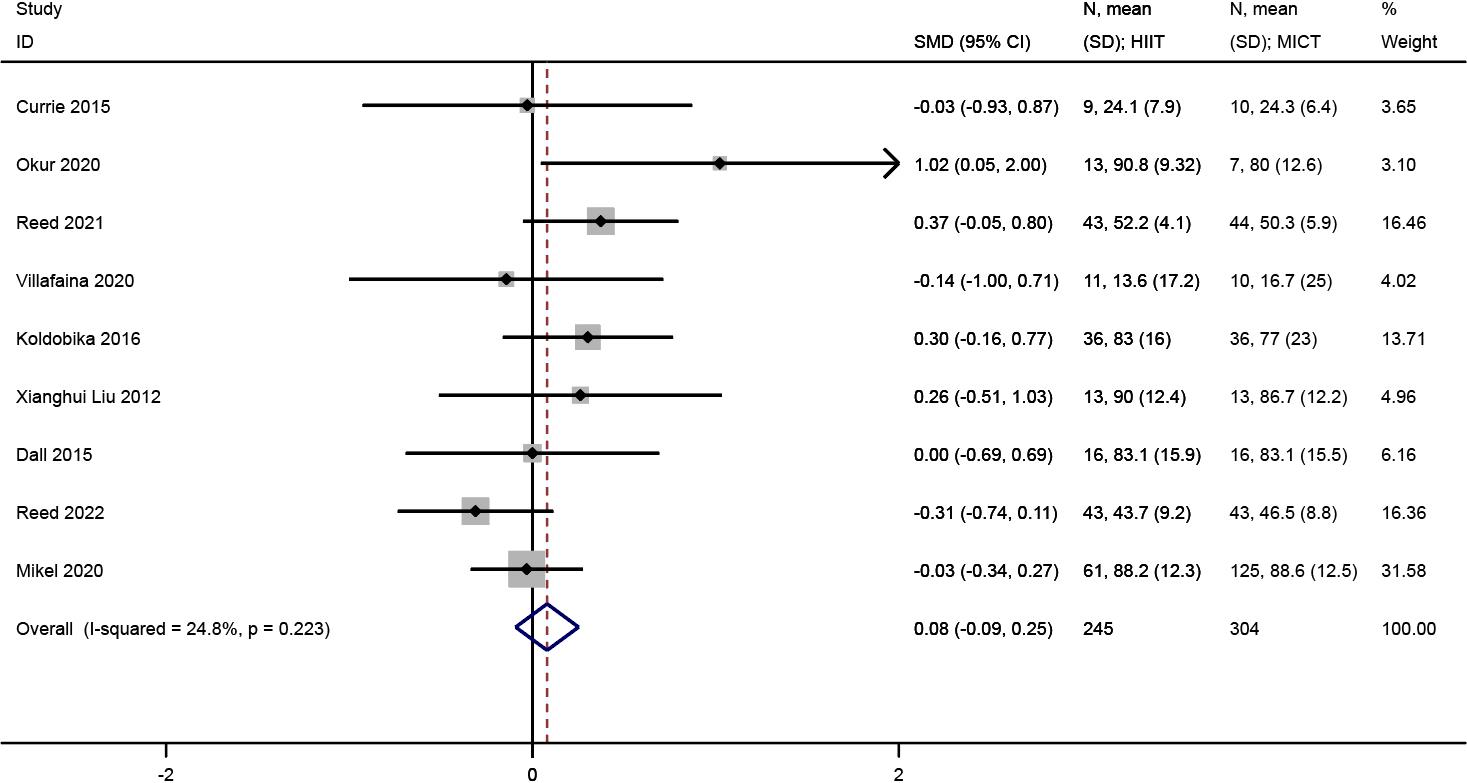


Physical functioning (PF)


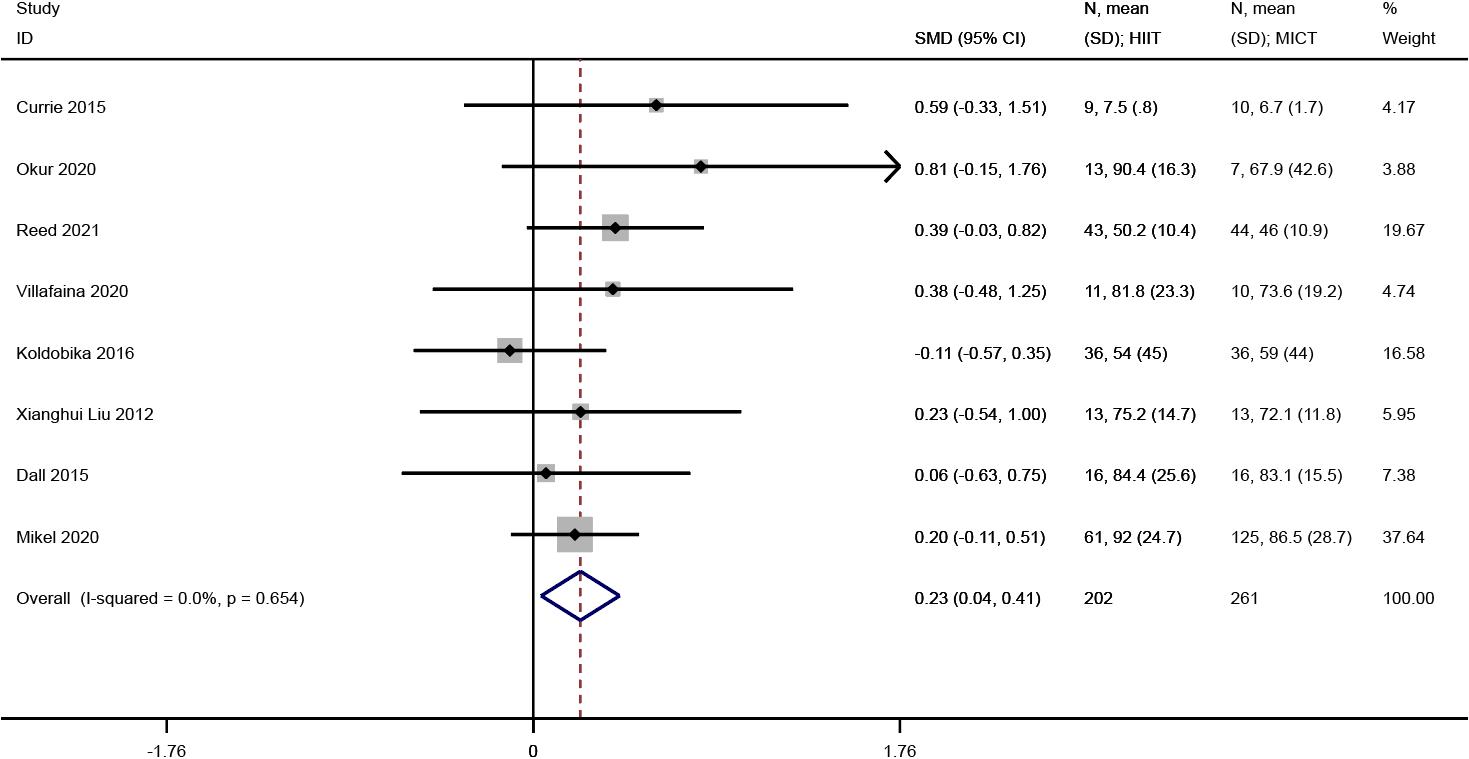


Role physical (RP)


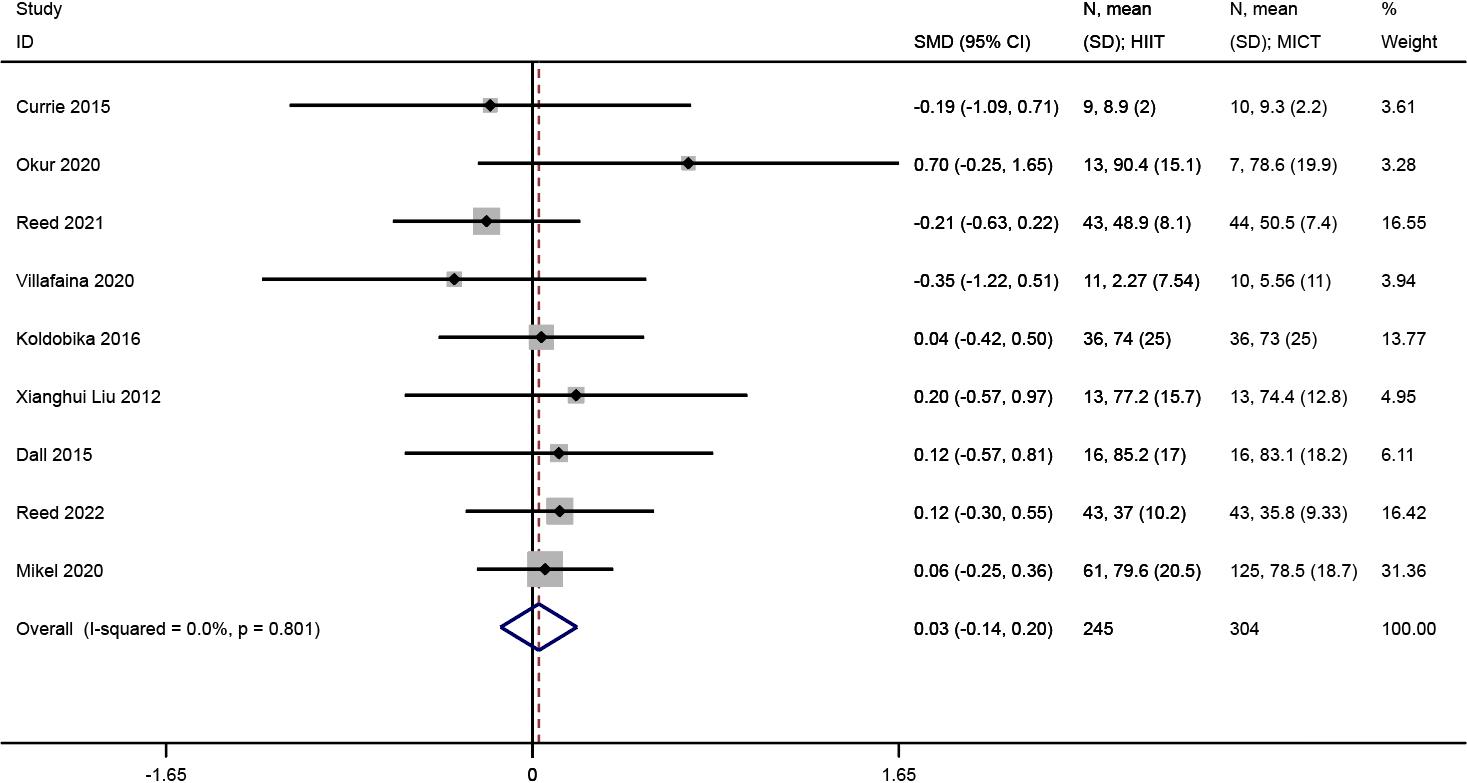


Bodily pain (BP)


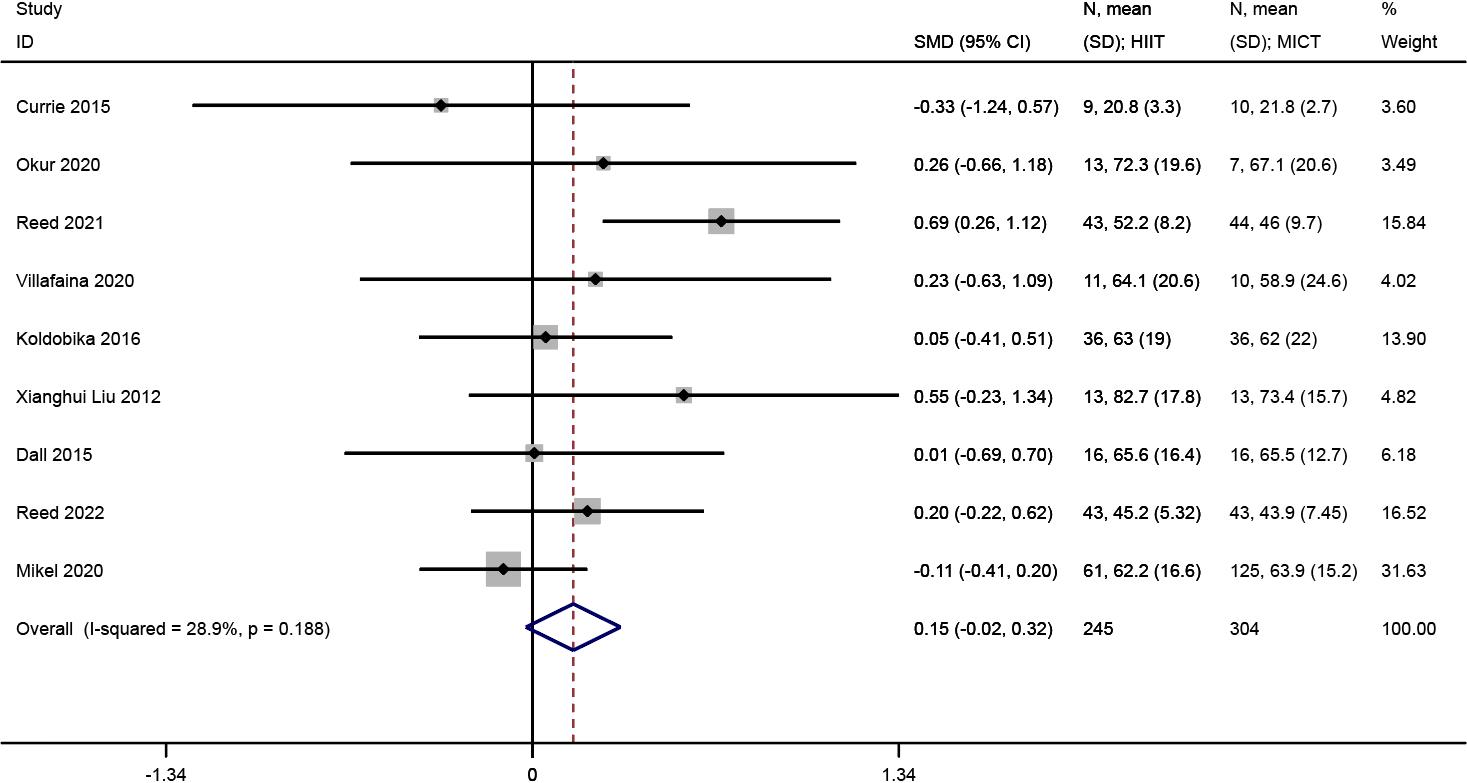


General health (GH)


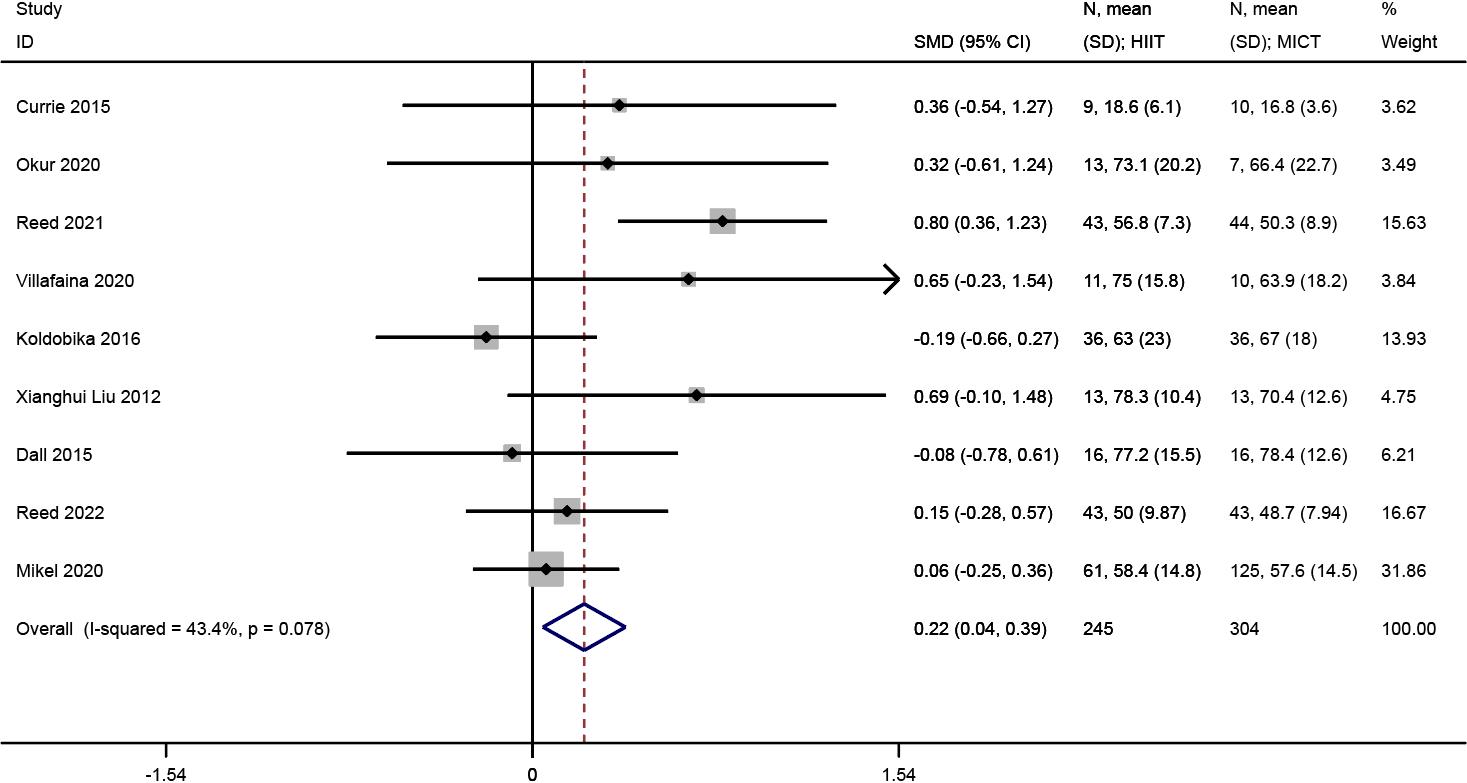


Vitality (VT)


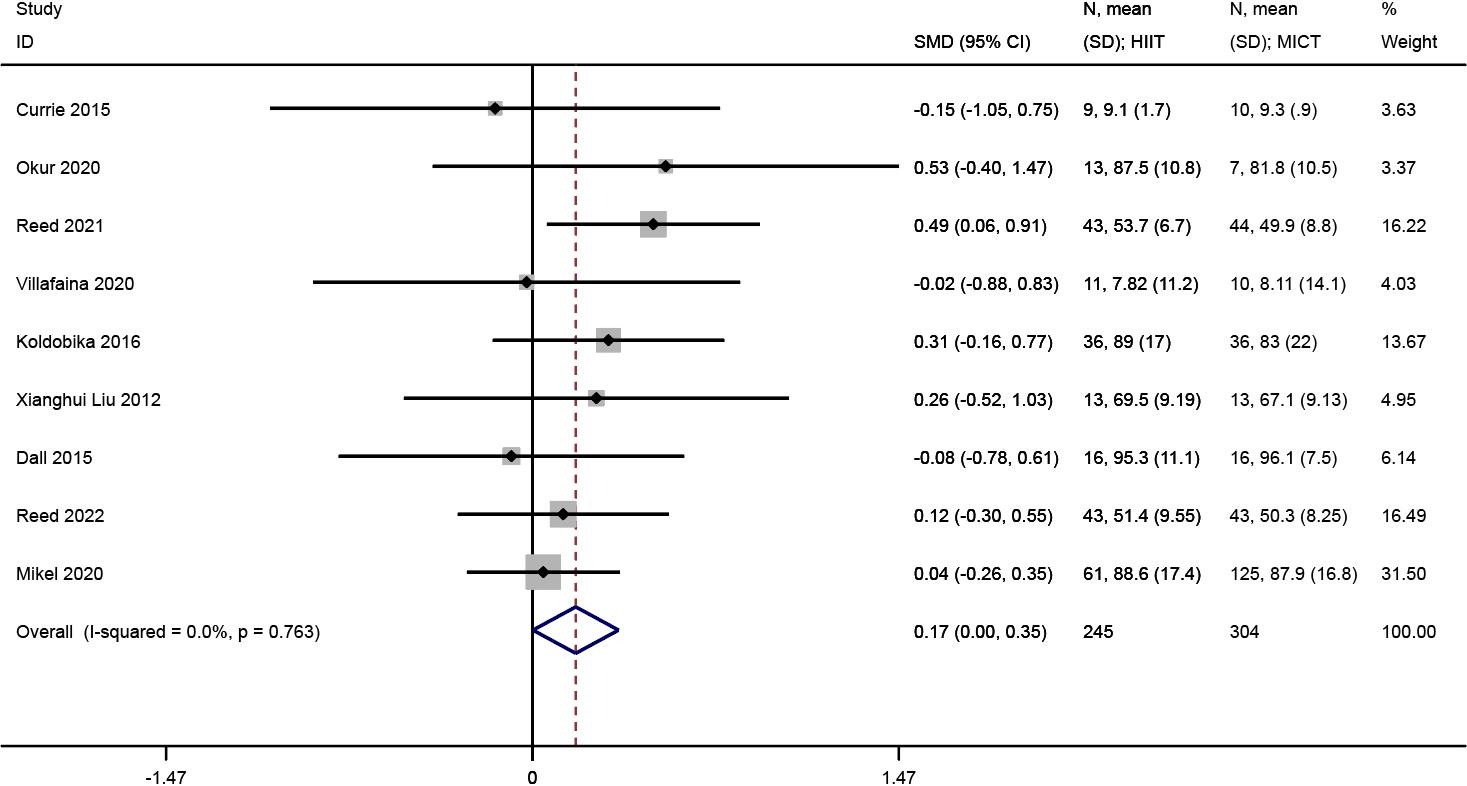


Social functioning (SF)


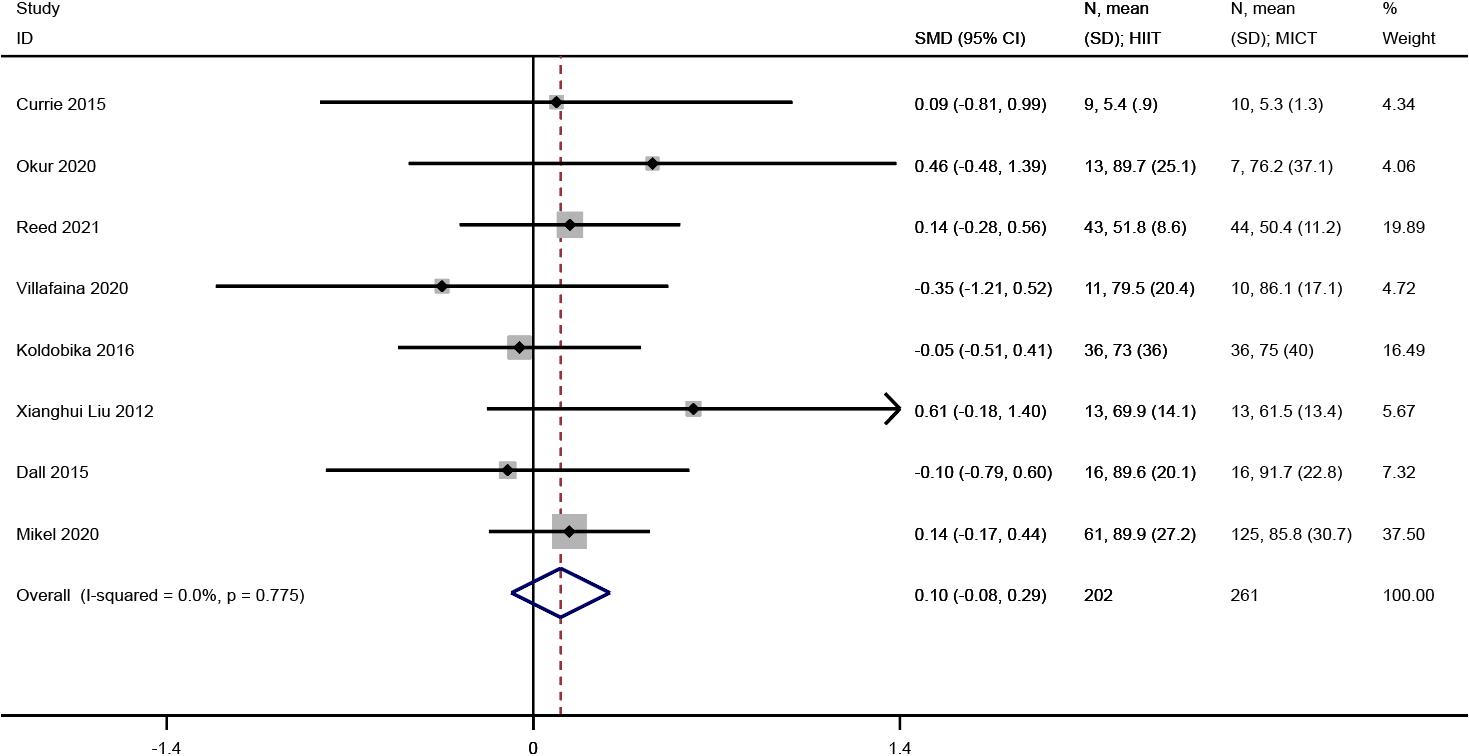


Role emotional (RE)


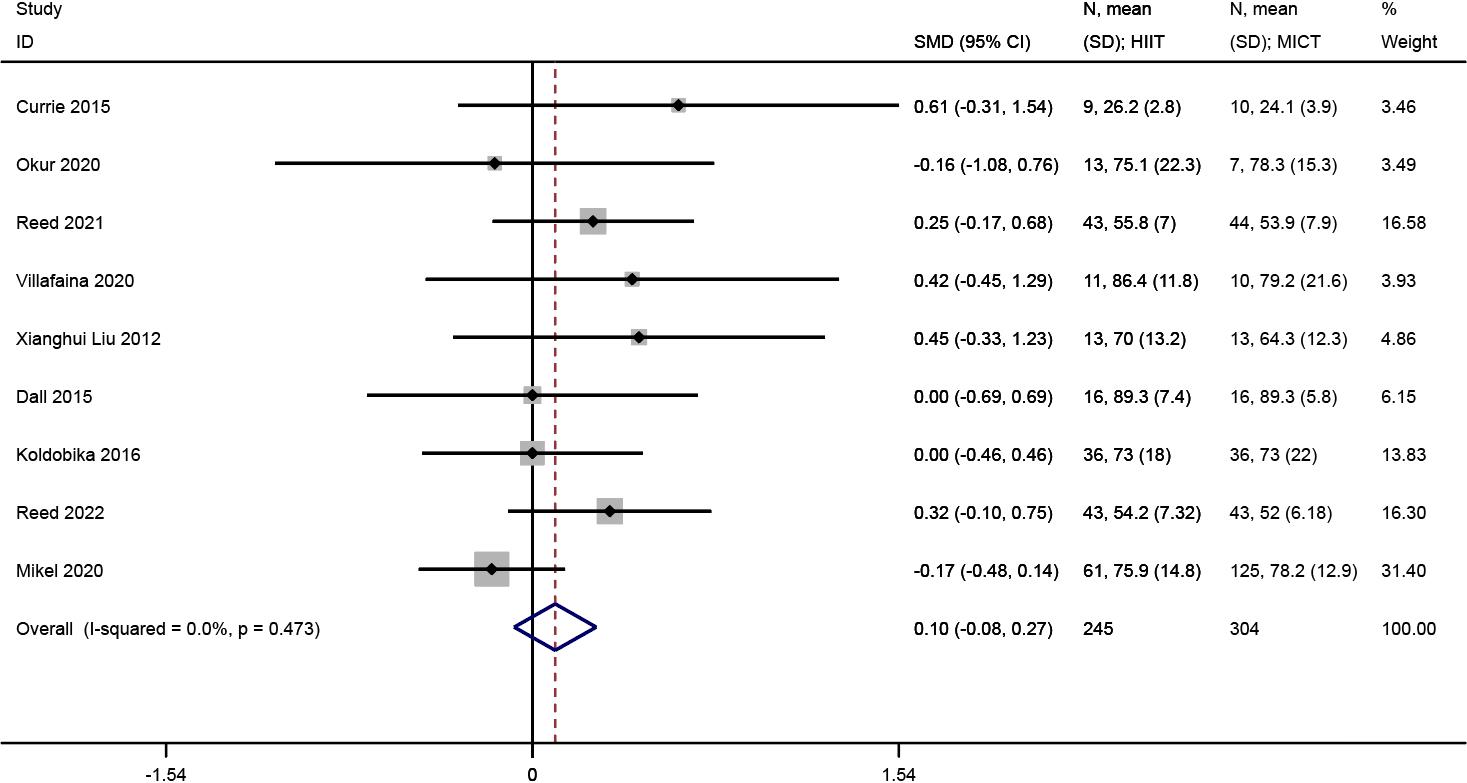


Mental health (MH)
