# Supplementary material for: High-intensity interval training versus moderate-intensity continuous training on patient quality of life in cardiovascular disease: a systematic review and meta-analysis
Source: Sci Rep. 2023 Aug 25;13:13915. doi: 10.1038/s41598-023-40589-5 (PMC10457360; doi:10.1038/s41598-023-40589-5)
Supplement: Supplementary file 5 — Supplementary Information 5. [file 41598_2023_40589_MOESM5_ESM.pdf]

Effects of High-Intensity Interval Training Versus Moderate-Intensity Continuous Training on mental health in adults: a systematic review and meta-analysis

Haohan Yu, Jingqi Hu, Ke Li, Lijuan Hou

To enable PROSPERO to focus on COVID-19 submissions, this registration record has undergone basic automated checks for eligibility and is published exactly as submitted. PROSPERO has never provided peer review, and usual checking by the PROSPERO team does not endorse content. Therefore, automatically published records should be treated as any other PROSPERO registration. Further detail is provided **here**.

Citation

Haohan Yu, Jingqi Hu, Ke Li, Lijuan Hou. Effects of High-Intensity Interval Training Versus Moderate-Intensity Continuous Training on mental health in adults: a systematic review and meta-analysis. PROSPERO 2022 CRD42022313051 Available from: https://www.crd.york.ac.uk/prospero/display_record.php?ID=CRD42022313051

Review question

Mental health is essential for maintaining health and well-being of individuals at all ages. High-Intensity Interval Training (HIIT) and Moderate-Intensity Continuous Training (MICT) are becoming more and more popular. Therefore, The objective of this systematic review is to synthesis the effects of High-Intensity Interval Training Versus Moderate-Intensity Continuous Training on mental health in adults.

Searches

We will search the following electronic bibliographic databases: MEDLINE, Embase, CINAHL, Cochrane Central Register of Controlled Trials (CENTRAL), Web of Science(Science and Social Science Citation Index), China Wanfang Database, China National Knowledge Infrastructure, China Science and Technology Journal Database. We will only include studies written in English and Chinese. No time restriction in the search. Moreover, all the references of the included articles will be screened to check whether any articles have been missed and thus need to be included.

Types of study to be included

We will include randomized controlled trials that examine the effects of High-Intensity Interval Training (HIIT) and Moderate-Intensity Continuous Training (MICT) on mental health. Papers based on single case studies, descriptive studies will be excluded. Literature reviews, systematic reviews, case reports, and opinion articles are also excluded. Only studies written in English and Chinese languages will be included.

Condition or domain being studied

Mental health is essential for maintaining health and well-being of individuals at all ages. While exercise training in adults has received accelerated attention, the best type, frequency, intensity and time of exercise still need to be explored. High-Intensity Interval Training (HIIT) and Moderate-Intensity Continuous Training (MICT) are becoming more and more popular. To the best of our knowledge, although previous randomized controlled trials have observed exercise, especially aerobic exercise to have beneficial effects on mental health, no quantitative meta-analysis based on experimental studies has been conducted about effects of HIIT and MICT on mental health in adults.

Participants/population

Inclusion: Adult participants (18 years or older).
Exclusion: Elderly people over 75

Intervention(s), exposure(s)

High-Intensity Interval Training (HIIT) and HIIT plus other exercises

Comparator(s)/control

Moderate-Intensity Continuous Training (MICT) and MICT plus other exercises

Context

There is no restriction on the setting (hospital, home, community, etc). And we will include exercise interventions with or without supervision.

Main outcome(s)

To describe the effects of HIIT and MICT on mental health in adults, such as mental well-being, depression, anxiety, sleep quality, etc.

Additional outcome(s)

To describe the effects of HIIT and MICT on health-related quality of life in adults.

Data extraction (selection and coding)

Two researchers will independently screen the titles and abstracts by the inclusion criteria. Then we will obtain full texts those titles and abstracts meet the inclusion criteria or where there is any uncertainty. Then two researchers will screen the full-text reports and distinguish whether it meets the inclusion criteria. If there are any disagreements, two researchers will make a discussion, or if unsuccessful, by consulting the senior researcher. Data extraction was performed by two researchers according to the data extraction table, details including (1) trial characteristics: country, design, sample size and so on; (2) participants' characteristics: age, gender and so on; (3) exercise intervention prescription: type, timing, frequency, follow-up, duration and so on.

Risk of bias (quality) assessment

We will use the Cochrane Collaboration's tool to assess each RCT study's risk of bias. It included adequacy of sequence generation, allocation concealment, blinding of participants and personnel, blinding of outcome assessment, incomplete outcome data, selective reporting, and other sources of bias. Two researchers working independently to assess the quality of the included studies. Disagreements were resolved by discussion, or if unsuccessful, by consulting the senior researcher.

Strategy for data synthesis

Data of included studies will be analyzed using Review Manager Software. Heterogeneity will be explored by the I^2 test. If the I^2 is less than 50%, a fixed-effects model will be employed. Otherwise, a random-effects model will be used. The effect size will be express by weighted mean difference (WMD) if the measurement and unit are the same in the studies. Otherwise, standardized mean difference (SMD) will be calculated. All data will be evaluated by 95% confidence intervals (CIs). If there are no applicable and substantial data to undertake a meta-synthesis, we will provide a narrative synthesis of the findings from the included studies, structured around the type of intervention, target population characteristics, type of outcome, and intervention content.

Analysis of subgroups or subsets

Subgroup analysis will be performed according to the heterogeneous factors considered.

Contact details for further information

Haohan Yu
vastime@outlook.com

Organisational affiliation of the review

College of P.E and Sports, Beijing Normal University, Beijing, China
https://english.bnu.edu.cn/

Review team members and their organisational affiliations

Mr Haohan Yu. College of P.E and Sports, Beijing Normal University, Beijing, China
Ms Jingqi Hu. College of P.E and Sports, Beijing Normal University, Beijing, China
Ms Ke Li. College of P.E and Sports, Beijing Normal University, Beijing, China
Professor Lijuan Hou. College of P.E and Sports, Beijing Normal University, Beijing, China

Type and method of review

Meta-analysis, Systematic review

Anticipated or actual start date

24 February 2022

Anticipated completion date

01 June 2022

Funding sources/sponsors

The National Natural Science Foundation of China

Grant number(s)
State the funder, grant or award number and the date of award
31971095

Conflicts of interest

Language

English

Country

China

Stage of review

Review Ongoing

Subject index terms status

Subject indexing assigned by CRD

Subject index terms

MeSH headings have not been applied to this record

Date of registration in PROSPERO

27 March 2022

Date of first submission

24 February 2022

Stage of review at time of this submission

Stage	Started	Completed
Preliminary searches	Yes	No
Piloting of the study selection process	No	No
Formal screening of search results against eligibility criteria	No	No
Data extraction	No	No
Risk of bias (quality) assessment	No	No
Data analysis	No	No

The record owner confirms that the information they have supplied for this submission is accurate and complete and they understand that deliberate provision of inaccurate information or omission of data may be construed as scientific misconduct.

The record owner confirms that they will update the status of the review when it is completed and will add publication details in due course.

Versions

27 March 2022
27 March 2022

PROSPERO
This information has been provided by the named contact for this review. CRD has accepted this information in good faith and registered the review in PROSPERO. The registrant confirms that the information supplied for this submission is accurate and complete. CRD bears no responsibility or liability for the content of this registration record, any associated files or external websites.
